# Systematic review and longitudinal analysis of implementing Artificial Intelligence to predict clinical deterioration in adult hospitals: what is known and what remains uncertain

**DOI:** 10.1093/jamia/ocad220

**Published:** 2023-11-14

**Authors:** Anton H van der Vegt, Victoria Campbell, Imogen Mitchell, James Malycha, Joanna Simpson, Tracy Flenady, Arthas Flabouris, Paul J Lane, Naitik Mehta, Vikrant R Kalke, Jovie A Decoyna, Nicholas Es’haghi, Chun-Huei Liu, Ian A Scott

**Affiliations:** Centre for Health Services Research, The University of Queensland, Brisbane, QLD 4102, Australia; Intensive Care Unit, Sunshine Coast Hospital and Health Service, Birtynia, QLD 4575, Australia; School of Medicine and Dentistry, Griffith University, Gold Coast, QLD 4222, Australia; Office of Research and Education, Canberra Health Services, Canberra, ACT 2601, Australia; Department of Critical Care Medicine, The Queen Elizabeth Hospital, Woodville, SA 5011, Australia; Eastern Health Intensive Care Services, Eastern Health, Box Hill, VIC 3128, Australia; School of Nursing, Midwifery & Social Sciences, Central Queensland University, Rockhampton, QLD 4701, Australia; Intensive Care Department, Royal Adelaide Hospital, Adelaide, SA 5000, Australia; Adelaide Medical School, University of Adelaide, Adelaide, SA 5005, Australia; Safety Quality & Innovation, The Prince Charles Hospital, Chermside, QLD 4032, Australia; Patient Safety and Quality, Clinical Excellence Queensland, Brisbane, QLD 4001, Australia; Patient Safety and Quality, Clinical Excellence Queensland, Brisbane, QLD 4001, Australia; School of Medicine and Dentistry, Griffith University, Gold Coast, QLD 4222, Australia; School of Medicine and Dentistry, Griffith University, Gold Coast, QLD 4222, Australia; School of Medicine and Dentistry, Griffith University, Gold Coast, QLD 4222, Australia; Centre for Health Services Research, The University of Queensland, Brisbane, QLD 4102, Australia; Department of Internal Medicine and Clinical Epidemiology, Princess Alexandra Hospital, Brisbane, QLD 4102, Australia

**Keywords:** clinical deterioration prediction, systematic review, AI implementation, machine learning, artificial intelligence, health informatics, digital health

## Abstract

**Objective:**

To identify factors influencing implementation of machine learning algorithms (MLAs) that predict clinical deterioration in hospitalized adult patients and relate these to a validated implementation framework.

**Materials and methods:**

A systematic review of studies of implemented or trialed real-time clinical deterioration prediction MLAs was undertaken, which identified: how MLA implementation was measured; impact of MLAs on clinical processes and patient outcomes; and barriers, enablers and uncertainties within the implementation process. Review findings were then mapped to the SALIENT end-to-end implementation framework to identify the implementation stages at which these factors applied.

**Results:**

Thirty-seven articles relating to 14 groups of MLAs were identified, each trialing or implementing a bespoke algorithm. One hundred and seven distinct implementation evaluation metrics were identified. Four groups reported decreased hospital mortality, 1 significantly. We identified 24 barriers, 40 enablers, and 14 uncertainties and mapped these to the 5 stages of the SALIENT implementation framework.

**Discussion:**

Algorithm performance across implementation stages decreased between *in silico* and trial stages. Silent plus pilot trial inclusion was associated with decreased mortality, as was the use of logistic regression algorithms that used less than 39 variables. Mitigation of alert fatigue via alert suppression and threshold configuration was commonly employed across groups.

**Conclusions:**

: There is evidence that real-world implementation of clinical deterioration prediction MLAs may improve clinical outcomes. Various factors identified as influencing success or failure of implementation can be mapped to different stages of implementation, thereby providing useful and practical guidance for implementers.

## Introduction

Clinical deterioration in hospitals is variously defined,^1^ most recently as, “An acute worsening of a patient’s clinical status that poses a substantial increase to an individual’s short-term risk of death or serious harm.”^2^^(p4)^ Clinical deterioration prediction algorithms based on machine or deep learning methods (herein called Machine Learning Algorithms—MLAs)^3^^,^^4^ present an opportunity to identify deteriorating patients earlier than existing rule-based methods^5–7^ such as the National Early Warning Score (NEWS),^8^ Modified Early Warning Score (MEWS),^9^ and Queensland Adult-Deterioration-Detection-System (Q-ADDS).^10^ Although MLA investigations are mostly retrospective *in silico* studies, many healthcare organizations are looking to implement MLAs into routine care to reduce mortality and morbidity. Retrospective analyses are of limited practical value when it comes to real-world implementation within health services which, according to theoretical frameworks, is a multistaged process with many factors influencing success.^11–15^ Health service decision-makers need to understand the enablers, barriers, and uncertainties that exist within end-to-end MLA implementation, from MLA selection based on retrospective validation studies through to prospective silent mode studies and live mode clinical trials and eventually routine use and postdeployment evaluation. In acquiring this understanding, a synthesis of published studies of clinical deterioration MLA implementation involving all stages of the process is needed in highlighting the differences in MLA implementation and their impacts on performance and clinical outcomes. Such a synthesis is presently lacking. Blythe et al^16^ reviewed studies on the clinical impact of implemented early warning systems that utilized real-time automated alerts, of which only 3 comprised MLAs. Three other reviews that focused on MLAs for clinical deterioration prediction included predominantly retrospective studies.^4^^,^^17^^,^^18^ For example, Muralitharan et al^4^ reported just 1 implemented system from 25 studies and Christodoulou et al^18^ reported none among 71 studies.

Mapping the various modes of implementation to a validated end-to-end Artificial Intelligence (AI) implementation framework^15^ further helps in identifying where and when enablers, barriers, and uncertainties apply to each stage of implementation. The SALIENT framework is stage-based and derived from authoritative clinical AI evaluation reporting guidelines^11^^,^^19–21^ in conjunction with Stead et al’s^22^ multistage approach to translating medical informatics interventions from the lab to the field. Compared to prior frameworks,^11–14^ SALIENT makes fully visible all components of the end-to-end solution, how and when they integrate, and the underlying implementation tasks. It has also been validated on real-world Sepsis prediction MLAs, similar to this work.^23^

In this study, we aimed to systematically review studies reporting the implementation or trialing of MLAs predicting clinical deterioration in adult hospitalized patients and map their findings to the SALIENT implementation framework.^15^

## Objectives

The first objective was to undertake a systematic review which identified and analyzed studies that implemented or trialed real-time clinical deterioration prediction MLAs. Analyses included (1) how MLA implementation was measured; (2) the impact of MLAs on clinical processes and patient outcomes; and (3) where and when barriers, enablers, and uncertainties apply within the implementation process. The second objective was to map the systematic review findings to the stages and elements of the SALIENT implementation framework.

## Materials and methods

### Search strategy

The systematic review was performed according to PRISMA guidelines.^24^ Five databases (PubMed/MEDLINE, EMBASE, Scopus, Web of Science, CINAHL) were searched between January 1, 2010 and April 1, 2023 for titles and abstracts published in English using keywords and synonyms for: (1) predict; AND (2) clinical deterioration; AND (3) machine learning; AND (4) trial; and NOT (5) child (see Appendix SA for complete search queries).

A forwards and backwards citation search (snowballing strategy) was then applied to identify additional articles reporting new MLAs, or, providing further information about MLAs described in previously included studies. The latter were labeled “linked” studies, describing the same MLAs at different stages of implementation, but not considered primary articles.

### Study selection

Studies of any design were included if: MLAs were applied to adult patients in hospital settings in whom clinical deterioration was identified; used live or near-live data; and reported at least one or more algorithm performance metric (full details in Appendix SB). Excluded studies were those not related to implementation or providing insufficient information for analysis. Covidence software^25^ supported a 2-stage screening process with screening of articles by 4 independent reviewers (A.H.V., V.R.K., P.J.L., and J.M.), with conflicts agreed by 3-way consensus (A.H.V., V.R.K., and P.J.L.); and full-text review by 3 independent reviewers (A.H.V., J.S., and T.F.), with selection agreed by 3-way consensus (A.H.V., J.S., and T.F.). Snowballing was then applied to all included studies and any new or linked studies were identified by A.H.V. and verified by J.A.D., N.E., and C.-H.L.

### Data extraction

Data were extracted independently by 4 authors (A.H.V., J.A.D., N.E., and C.-H.L.) using Excel templates, with disagreements resolved by consensus. Extracted data included study metadata, implementation stage, care setting, MLA details including training and validation datasets, performance metrics, outcome definitions and events (including mortality, cardiac arrest and unplanned transfer to intensive care units [ICUs]), and implementation barriers, enablers, and uncertainties (see Appendix SC for more details). Barriers were defined as pitfalls or problems hindering implementation success; enablers as tips or activities aiding implementation success. Uncertainties were identified when 2 or more studies chose different approaches for the same implementation decision. Consensus between authors (A.H.V., J.A.D., N.E., and C.-H.L.) determined which individual barriers, enablers, and uncertainties to include and which to consolidate under a common title to minimize overlap.

### Mapping to AI implementation framework

**Figure 1.**
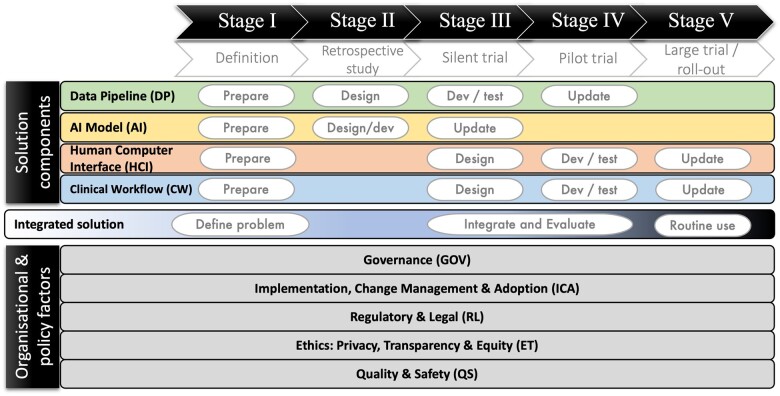
Abridged version of the SALIENT clinical Artificial Intelligence (AI) implementation framework.^23^ The stages of implementation (I-V) are listed across the top in black and white, with a short description of each stage beneath. The AI solution components are provided in 4 bars, labelled on the left hand side that stretch across each stage. Key implementation tasks are identified in white boxes within each component underneath the stage they are likely to occur in, including: preparation, design, development and testing (dev/test), and update. The integrated solution is represented by the bar underneath the solution components. Tasks within the integrated solution are the problem definition in stage I, integration and evaluation of the solution in stages III and IV, and then routine use in stage V. Other crossstage organizational and policy factors are provided as 5 bars at the bottoms of the diagram.

The systematic review findings for each barrier, enabler, and uncertainty were mapped to at least 1-stage and 1-solution component or organization and policy factor within the SALIENT implementation framework (Figure 1). SALIENT element descriptions are provided in Table 1. Mapping was followed by a review by A.H.V. and V.C. and adjustments were made where discrepancies were found.

**Table 1.** Reference code (column 1), name (column 2), and descriptions (column 3) of each SALIENT stage, Artificial Intelligence (AI) solution component and organization, and policy factor (column 2) that barriers, enablers, and uncertainties were mapped to.

	SALIENT element	Element description
Stages of implementation		
I	Definition	When the clinical problem is defined, the rationale for change, background, context, and intended use of the potential Artificial Intelligence (AI).
II	Retrospective study	When a retrospective, *in silico* evaluation is performed on the AI algorithm solution component.
III	Silent trial	When a prospective, live-data evaluation is performed on the AI algorithm and data pipeline solution components. Also called a silent or shadow trial.
IV	Pilot trial	When a small trial is conducted within clinical practice to evaluate the whole AI solution and to identify issues and problems before moving to a larger trial or roll-out of the solution.
V	Large trial/roll-out	When the solution is run in its operational environment, within clinical practice and evaluated as a larger trial, such as a random controlled trial, or as a general roll-out across hospital wards.
Implemented solution components		
DP	Data pipeline	The technology and infrastructure extending from where real-time clinical data is captured, stored, extracted, transferred, and transformed to where it is made available for use by the AI model and human-computer interface.
AI	AI model	The MLA development, training, and deployment including any the algorithm employed, variables used as input and any configuration and tuning.
HCI	Human-computer interface	The user interface (eg, dashboard) or mechanism employed (eg, mobile alert) to transfer the outputs of the AI model to the clinician. Includes content, layout, format, and interactivity.
CW	Clinical workflow	The changes required to the existing clinical workflow that are designed to accommodate the AI model outputs and human-computer interface.
Integrated solution		
		This complete solution that integrates the system components (data pipeline, AI model, and human-computer interface) with the new clinical workflow. After integration the solution is evaluated before moving to routine use
Organization and policy factors		
GOV	Governance	The governance of all aspects of implementation including the scope of the solution, model selection process, and the extent of oversight required.
ICA	Implementation, change management, and adoption	The management of the implementation projects including identification of stakeholders, leadership, implementation roles and responsibilities, change process, and solution adoption approach
RL	Regulatory and legal	The legal regulatory approval and compliance process for deploying AI solutions and other legal factors, such as legal responsibility and accountability
ET	Ethics	The ethical aspects of implementing an AI solution including patient data privacy, cyber-security, transparency of the use of AI and interpretability of its outputs, auditability, equity of AI use including bias and fairness considerations
QS	Quality and safety	The solution quality and safety considerations including patient risk, incident reporting and monitoring and maintenance of quality and safety indicators

### Quality assessment

Studies reporting hospital mortality underwent a risk of bias (RoB) assessment as mortality was the most frequently reported patient outcome measure and considered the most important. RoB assessment was performed independently by 2 authors (A.F. and N.M.), using the ROBINS-I tool^26^ for nonrandomized studies and the Cochrane RoB 2 tool^27^ for randomized studies.

## Results

**Figure 2.**
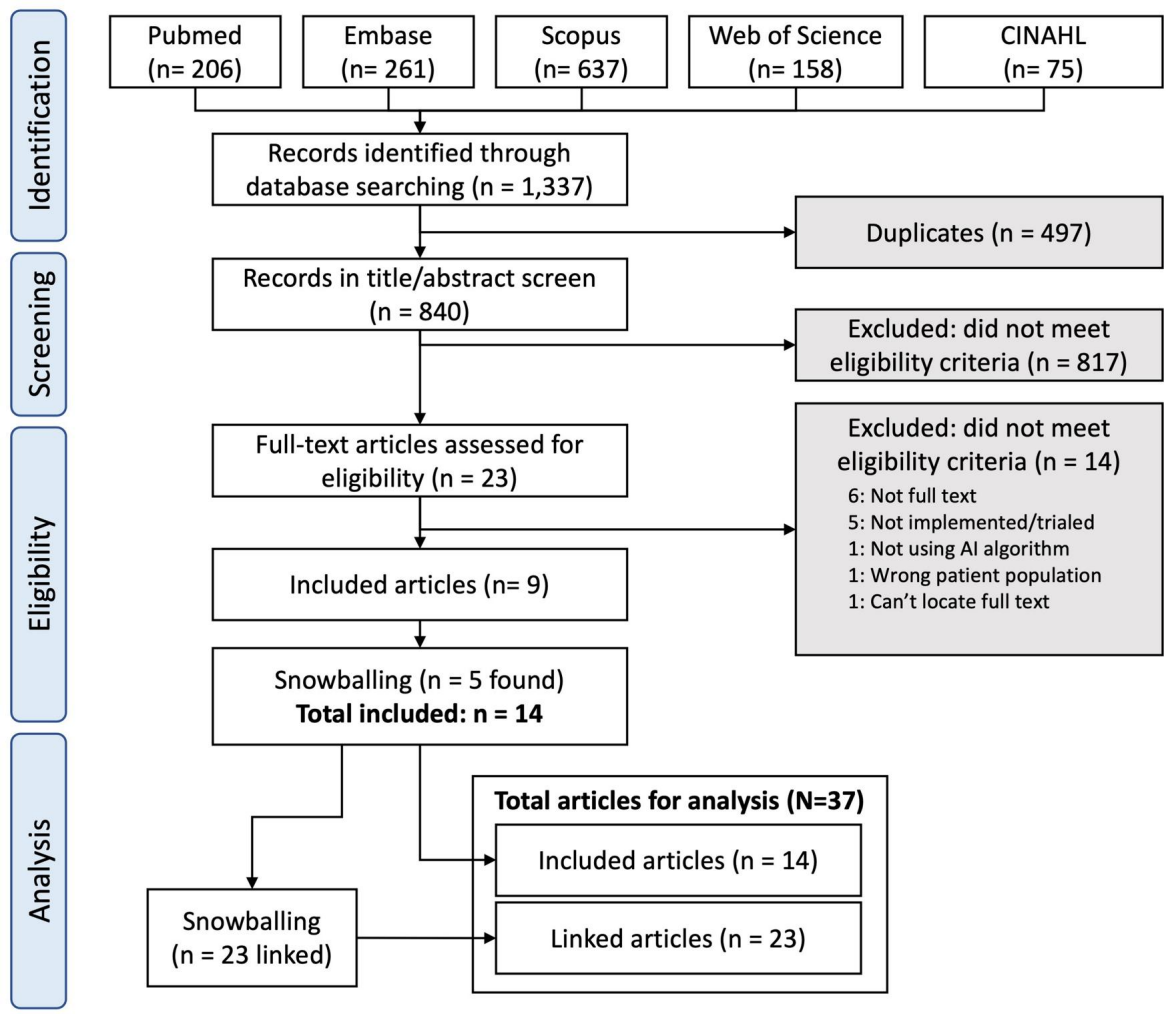
PRISMA-ScR flowchart for study selection.

From 1337 retrieved abstracts, 497 duplicates were removed, leaving 840 for screening, from which 9 full-text studies were included for analysis (Figure 2).^28–36^ Most excluded studies were not full text or not implemented. An additional 5 articles found by snowballing were selected,^37–41^ yielding 14 studies as primary articles, with further snowballing yielding 23 linked studies,^6^^,^^7^^,^^42–62^ giving a total of 37 articles for analysis.

### Study characteristics

The 37 studies were published between 2011 and 2023, with 14 algorithm groups (A to N) identified according to the common or named MLA that was the focus of study (Table 2); 10 were US-based (A, C, E-I, K, L, N) with one group each from Australia (B), Korea (D), Canada (J), and Singapore (M). Six groups (A, F, G, H, K, M) implemented live mode MLAs with a quantitative evaluation (before-after study,^55^^,^^60^ randomized controlled trial,^28^^,^^44^ controlled trial,^40^ difference-in-difference study,^38^ cohort study^36^). Seven groups (A, B, C, F, I, L, N) conducted silent trials with quantitative evaluations (prospective evaluation,^29^^,^^31^^,^^33^^,^^35^^,^^37^^,^^41^ simulation^42^). Two groups (H, J) conducted qualitative case studies during^58^^,^^61^^,^^62^ or after^32^^,^^47^^,^^51^^,^^54^ live mode implementation and one group (M) prior to implementation.^56^ Three groups (D, E, L) reported postimplementation retrospective studies.^30^^,^^39^^,^^49^ All except 3 groups reported retrospective *in silico* studies validating the MLA prior to implementation (L, M, N).

**Table 2.** Study characteristics.

Group (MLA name)	Algorithm	Reference	SALIENT stage	Study design (no. of sites)	Outcome	Outcome count (prevalence) (%)	AUC	Sensitivity (%)	PPV (%)
A USA; Uni of Washington	Log. regression 36 variables	Mao et al (2011)^48^ Hackmann et al (2011)^42^ Hackmann et al (2011)^42^ Bailey et al (2013)^28^ Bailey et al (2013)^28^ Kollef et al (2014)^44^	II II III II IV IV	R(1) R(1) S(1) R(1) RCT(1) RCT(1)	icu icu icu, D icu, D icu, D	1173 (4.1) 1173 (4.1) 1173 (4.1) 1320 (3.4) 146 (26)	0.92 0.88 0.73 0.88	61 49 41 41-54	38 31 30 10-15
B Aust; Ainsoft Inc.	Log. regression 43 variables	Bell et al (2021)^29^ Bell et al (2021)^29^	II III	R(2) P(1)	icu, D, met, uot for all	802 (3.4) 407 (1.4)	0.92 0.89	41 27-39	
C USA, Duke Uni.	XGBoost 57 variables	Brajer et al (2020)^37^ Brajer et al (2020)^37^	II III	R(2) P(1)	D D	203 (1.9) 84 (1.6)	∼0.87 0.86	60 60	8-9 9
D (DEWS) Korea; Vuno Inc.	LSTM 4-7 variables	Kwon et al (2018)^45^ Cho et al (2020)^39^ Lee et al (2021)^46^	II Post II	R(2) R(1) R(3)	ca icu, ca ca	23 (0.4) 83 (1.0) 201 (0.1)	∼0.85 0.87 0.91	37 51 34	4 3 2
E (APPROVE) USA, Mayo et al	Random forest 36 variables	Dziadzko et al (2018)^30^ Dziadzko et al (2018)^30^	II Post	R(4) R(1)	D, mv D, mv	2030 (3.0) 35 (1.5)	0.87 0.90	0.63 0.64	21 16
F (eCart) USA, Uni of Chicago, AgileMD Inc.	Log. regression 27-33 variables	Churpek et al (2014)^57^ Churpek et al (2014)^6^ Kang et al (2016)^31^ Bartkowiak et al (2019)^52^ Winslow et al (2022)^55^	II II III II V	R(1) R(4) P(1) R(1) BA(3)	ca ca icu, ca icu, D, ca icu	109 (0.2) 160 (0.2) 393 (7.1) 1243 (3.8) 6930 (12)	0.88 0.83 0.80 0.79	60-77 54 53-100 75	
G (MEWS++) USA, Icahn Sch.	Random forest 36 features	Kia et al (2020)^7^ Levin et al (2022)^40^	II IV	R(1) CT(1)	icu, D icu, D	1997 (3.4) 292 (10.5)	0.88	79	12
H (AAM) USA, Kaiser Permanente	Log. regression 39 variables	Escobar et al (2012)^59^ Kipnis et al (2016)^43^ Dummett et al (2016)^58^ Escobar et al (2016)^61^ Granich et al (2016)^62^ Escobar et al (2020)^60^ Lisk et al (2020)^47^ Paulson et al (2020)^51^ Martinez et al (2022)^32^	II II IV IV IV V Post Post Post	R (14) R (21) CS (2) CS (2) CS (2) BA(19) CS (19) CS (19) CS (19)	icu, D icu, D icu, D	4036 (9.2) 19 153 (2.9) 36 233(6.6)	0.78 0.82	49 25	8 16
I USA, Duke University	Log. regression 50 variables	O’Brien et al (2019)^33^ O’Brien et al (2019)^33^	II III	R (1) P (1)	icu, D icu, D	(3.7) 130 (3.1)	0.81 0.75	5	10
J (CHARTwatch) Canada, Vector Institute	Log. regression and ensemble 526 variables	Nestor et al (2020)^50^ Verma et al (2021)^54^ Pou-Prom et al (2022)^34^ Pou-Prom et al (2022)^34^	II Post II IV	R (1) CS (1) R (1) CS (1)	icu, D, ca icu, D icu, D	1186 (9.6)	∼0.95 0.63 0.75	40 48 57	71 17 26
K (MC-EWS) USA, Mayo Clinic	Gradient boosting 59 variables	Romero-Brufau et al (2021)^38^^,^^53^	II II IV	R (2) R (1) DiD(1)	icu, met, ca icu, met, ca	1547 (4.1) 6909 (12) 933(∼8.0)	0.91 0.94	73	12
L (EPIC EDI) USA	Log. regression >125 variables	Singh et al (2021)^35^ Mou et al (2022)^49^	III Post	P(1) R(1)	icu, mv, D D	103 (26)^a^ 27 (2.0)	0.79 0.91	39 93	74 23
M (Biovitals) Singapore, Bioformis Inc.	Linear vector regression;	Chen et al (2019)^56^ Un et al (2020)^36^	I IV	CS O	icu, various	17 (50)	0.93	94	89
N USA, HBI Solutions Inc.	Random forest 349 variables	Ye et al (2019)^41^	III	P(2)	D	255 (2.2)	0.88	23	31

Abbreviations: P, prospective; R, retrospective; S, simulation; O, observational; CT, clinical trial; RCT, randomized controlled trial. Outcomes: D, death; icu, unplanned transfer to ICU; ca, cardiac arrest; met, medical emergency team call; uot, unplanned return to operating theatre; mv, mechanical ventilation; NR, not reported.

a Subcohort of covid patients only.

Groups (F, H) included the only multicenter trials^55^^,^^60^ and group H included the only trial reporting more than 10 000 outcome events.^60^ Median silent trial length was 3.5 months (IQR 3.5) and live clinical trial length was 9 months (IQR 10.5).

The prevalence of deterioration outcomes varied from as low as 2.1%^63^ to as high as 22.7% in non-ICU settings,^64^ and from 11.3%^65^ to 32.8%^66^ in ICU settings.

Eighteen studies reported MLA evaluations at SALIENT implementation stage II (retrospective validation),^6^^,^^7^^,^^28–30^^,^^33^^,^^34^^,^^37^^,^^42^^,^^43^^,^^45^^,^^46^^,^^48^^,^^50^^,^^52^^,^^53^^,^^57^^,^^59^ 6 at stage III (silent trial),^29^^,^^31^^,^^33^^,^^35^^,^^37^^,^^41^ 6 at stage IV (pilot trial),^28^^,^^34^^,^^36^^,^^38^^,^^40^^,^^44^ 2 at stage V (large trial/roll-out),^55^^,^^60^ and 3 reported postdeployment evaluations.^30^^,^^39^^,^^49^

### Quality assessment

Six publications from 5 groups (A, F, G, H, K) were assessed for RoB (see Appendix SD). Overall, RoB was serious for 2 groups (G, K) and moderate for 3 (A, F, H). Major sources of bias, which no trial controlled for,^38^^,^^55^^,^^60^ were potential confounders from additional alert system cointerventions, such as trial staff involvement in the design and setup of the MLA within clinical workflows and special training and different patient care protocols associated with new clinical workflows, all potentially impacting hospital mortality and clinical process indicators at the trial sites.

### Implementation and clinical impact evaluation

One hundred and seven distinct metrics were identified across 33 (89%) studies, grouped into 4 evaluation categories: (1) algorithm performance; (2) alert performance; (3) clinical process effects; and (4) patient outcome effects. All metrics reported are listed in Appendix SE.

#### Algorithm and alert performance

Of 33 algorithm performance metrics, sensitivity and area under the receiver operating curve (AUROC) were reported across all groups, with positive predictive value (PPV) (12 groups) and specificity (11 groups) the next most common. Most algorithm metrics (70%) were common to at-most 2 groups. Alert metrics (*n* = 20) were reported by 79% (*n* = 14) of groups, with 8 reporting median or average alert hours before the deterioration event. All other metrics were common to at-most 2 groups, however, 12 of the remaining 18 alert metrics reported were a variant of the mean alarm count per day (MACD).

All of 6 studies that evaluated stage III (silent) trials reported algorithm performance metrics^29^^,^^31^^,^^33^^,^^35^^,^^37^^,^^42^ and 4 of these also reported stage II (retrospective) results^29^^,^^33^^,^^37^^,^^42^; algorithm performance declined in all 4 between stages II and III for at least one of AUROC, sensitivity, or PPV. Only one study reported algorithm performance at both stage III (silent) and IV/V (Trial/roll-out), also reporting a decrease in AUROC.^34^ Dziadzko et al is the only study reporting comparable stage II and postimplementation algorithm performance in which AUROC improved (0.87-0.90) for a very small sample (35 patient outcomes). However, PPV fell by 24% affirming that AUROC stability across settings is only one marker of MLA quality.

#### Clinical impact

A total of 37 clinical process metrics, defined as measures of impact on clinical practice, were reported within 33 studies, 17 solely reported by Kollef et al,^44^ who evaluated a range of diagnostic and therapeutic interventions administered within 24 h of the alert, including antibiotics, vasopressors, and oximetry. Of the 20 remaining metrics, those reported by more than one group were ICU transfer rates (5 groups, 6 studies) and median hours between alert and clinical escalation (2 groups, 2 studies).

Seventeen patient outcomes were reported, the most common being hospital mortality (5 groups, 6 studies), hospital length of stay (LOS) (3 groups, 4 studies), and ICU LOS and 30-day mortality (both 2 groups, 2 studies).

Table 3 reports algorithm performance by SALIENT stage of implementation and clinical impact for the 5 groups reporting in-hospital mortality. Four of the 6 studies reporting hospital mortality showed numerical improvement,^40^^,^^44^^,^^55^^,^^60^ of which Winslow et al^55^ (group F) reported the only statistically significant reduction. Groups G and H also reported statistically significant reduction in mortality, but for combined in-hospital and 30-day mortality (G) and death within 30 days of first alert (H). All 4 also reported improved clinical process metrics, 3 being statistically significant (groups A, F, G).^40^^,^^44^^,^^55^ Although group G did not report a statistically significant reduction in hospital mortality alone, it did report a 2.5% statistically significant reduction in the combined metric of in-hospital and 30-day mortality. Groups A and F were the only groups to report stage III (silent trial) algorithm performance with AUROC of 0.73 and 0.80, respectively. Two studies reported no change or statistically insignificant hospital mortality.^28^^,^^53^ The largest study (group H, 36 233 outcomes^60^) reported nonsignificant improvement in clinical process metrics and mortality.

**Table 3.** Evaluation results for each group that reported in-hospital mortality before and after the implementation of the MLA.

			MLA evaluation results by SALIENT stage								Clinical impact						
		Study outcome count (prevalence %)	Stage II Retrospective			Stage III Silent trial			Stage IV/V Post-impl.		Clinical processes				Mortality change		
Group and study			AUC	Sensitivity (%)	PPV (%)	AUC	Sensitivity (%)	PPV (%)	Sensitivity (%)	PPV (%)	No. of improve	No. of improve*	No. of decline	No. of decline*	In-hospital	Other	Risk of bias
A	Mao et al (2011)^48^	1173 (4.1)	0.92	61	38												
	Hackmann et al (2011)^42^	1173 (4.1)	0.88	49	31												
	Hackmann et al (2011)^42^					0.73	41	30									
	Bailey et al (2013)^28^	1320 (3.4)							41-54	10-15					0		
	Kollef et al (2014)^44^	146 (26)									1	2		1	]		M
F	Churpek et al (2014)^57^	109 (0.2)	0.88	60-77													
	Churpek et al (2014)^6^	160 (0.2)	0.83	54													
	Kang et al (2016)^31^	393 (7.1)	0.80	53-100		0.8	53-100										
	Bartkowiak et al (2019)^52^	1243 (3.8)	0.79	75													
	Winslow et al (2022)^55^	6930 (12)										6			]*		M
G	Kia et al (2020)^7^	1997 (3.4)	0.88	79	12												
	Levin et al (2022)^40^	292 (10.5)		93	7						7	1			]	]*^,a^	S
H	Escobar et al (2012)^59^	4036 (9.2)	0.78		8												
	Kipnis et al (2016)^43^	19 153 (2.9)	0.82	49	16												
	Escobar et al (2020)^60^	36 233(6.6)									3				]	]*^,b^	M
K	Romero-Brufau et al (2021)^38^^,^^53^	1547 (4.1)	0.91														
		6909 (12)	0.94	73	12												
		933(∼8.0)										2	1		Ζ		S

* Results for each group study include: (1) the outcome count and percent prevalence; (2) the SALIENT stage II, III, and combined IV/V MLA evaluation results, reported as area under the receiver operating curve (AUC), sensitivity, and positive predictive value (PPV); (3) the clinical process improvements, measured as the number of improved processes and the number of processes that declined ( represents significant change); (4) the mortality change (in-hospital and other) where ],Ζ,0 indicate decrease, increase, no change and where trailing

* indicates significant result; and (5) the risk of bias assessment for the study reporting mortality outcomes.

a Combined in hospital and 30-day mortality.

b Death within 30 days of first alert.

### Implementation factors and mapping to SALIENT framework

#### Barriers and enablers

We identified 24 barriers and 40 enablers from a total of 225 mentions across all studies. Tables 4 and 5 list the barriers and enablers as identified by at least 2 groups. The most common barriers (ie, those identified by at least 4 groups) were limitations in Electronic Health Record (EHR) data (B1), ICU transfer as a poor outcome for MLA training and evaluation (B2), alert fatigue (B3), EHR data entry delays (B4), and site-by-site prevalence differences in deterioration outcomes requiring MLA retraining (B5). Nine barriers (38%) were each found in just one group. The median barrier mentions per group was 3, with group H accounting for 25 (35%) and groups (I, M) contributing none.

**Table 4.** Implementation barriers reported by at least 2 groups (see Table SF1 for full listing).

Group count (%)	Study count (%)	ID	Barriers (SALIENT stage)	SALIENT component or element
7 (58)	8 (27)	B01	Inherent limitations of EHR data, which can be plagued by missingness, inaccuracies, and changes in practice patterns over time; Manually collected vital sign readings resulting in very irregular time series and multiscale gaps. (II+)	DP; AI
5 (42)	8 (27)	B02	Use of ICU transfer is not a good outcome for Artificial Intelligence (AI) development as admission to ICU criteria may differ between hospitals. (II/III)	AI; EV
4 (33)	5 (17)	B03	Alert fatigue (IV/V)	AI; CW; ICA
4 (33)	5 (17)	B04	Data entry delays, leading to delayed predictions (III+)	DP
4 (33)	4 (13)	B05	Differences in outcome prevalence at different sites might require models to be retrained or at least new alert thresholds selected for those sites to maintain target PPV. (V)	AI
3 (25)	4 (13)	B06	Lack of clinician trust. (IV+)	ICA
3 (25)	4 (13)	B07	Lack of a specific and/or effective action for the clinician to take when alerted; Differential nurse/doctor role or perceptions of role and value; Variations in hospital governance for defining standardized response processes. (IV+)	CW; ICA; GOV
3 (25)	3 (10)	B08	Lack of infrastructure to produce live EHR data pipelines. (III+)	DP
3 (25)	3 (10)	B09	Major differences between retrospective data elements and prospective/trial data elements. (III)	DP; AI
3 (25)	5 (17)	B10	Hard/impossible to assess in an implementation study whether a lack of clinical outcome is due to the algorithm performance or the downstream Rapid Response Team (RRT) system; Conducting RCT are often not possible. (IV+)	EV
3 (25)	3 (10)	B11	Insufficient event samples to build a model that incorporates patient subgroups; biases in algorithm for different patient groups. (II)	AI; ethics; QS
2 (17)	2 (7)	B12	Substantial cost involved for infrastructure, implementation personnel time, and ongoing maintenance. (II+)	ICA; GOV
2 (17)	2 (7)	B13	Lack of individual proficiency of health professionals in the use of hardware and software. (IV+)	CW; ICA
2 (17)	2 (7)	B14	No measurement of whether the alerted physician followed through with an action. (IV+)	EV
2 (17)	3 (10)	B15	Differences in software versions between research and production environments. (IV+)	DP; AI

Includes the number and percentage (*n* = 12) of groups (column 1) and the number and percentage (*n* = 30) of studies (column 2) reporting each barrier. The last column contains the mapping to SALIENT components and elements. Salient components are: HCI, human-computer interface; AI, artificial intelligence model; CW, clinical workflow; DP, data pipeline. SALIENT elements are: ICA, implementation, change management and adoption; EV, evaluation; RL, regulatory and legal; QS, quality and safety; Ethics, privacy, transparency and equity; GOV, governance.

**Table 5.** Implementation enablers reported by at least 2 groups (see Table SF2 for full listing).

Group count (%)	Study count (%)	ID	Enablers (SALIENT stage)	SALIENT component or element
8 (61)	16 (45)	E01	Clinician involvement essential at all stages of model/HCI development and integration into clinical workflow. (II+)	AI; CW; HCI; GOV
6 (46)	13 (37)	E02	Methods identified to reduce false alarms and alert fatigue. (II+)	AI; CW; HCI
5 (38)	10 (28)	E03	Linking the EWS alert to specific clinician actions. Clarifying clinical decision points, who is responsible and the actions to take. (III/IV)	CW; RL
5 (38)	7 (20)	E04	Using more EHR variables than just vital signs can improve accuracy. (II)	AI
4 (30)	6 (17)	E05	Establishing a transdisciplinary team of data scientists, statisticians, hospitalists, intensivists, ED clinicians, RRT nurses, and information technology leaders and developing capabilities across domains. (I+)	ICA; GOV
4 (30)	5 (14)	E06	Conducting external validations using datasets different in both time and geographical location may support models that require less updates and retraining. (II)	AI
4 (30)	5 (14)	E07	Providing additional data with the alert for clinicians to help contextualize the information. (III+)	HCI
3 (23)	7 (20)	E08	Frequent communications to increase awareness during and after trial, for example, weekly meetings, emails, educational sessions giving progress reports and setting next goals and highlighting urgent need. (IV+)	ICA
3 (23)	5 (14)	E09	Iterative approach to design of clinical workflow, human-computer interface (HCI), and MLA model. (II+)	AI; CW; HCI
3 (23)	5 (14)	E10	Performing postimplementation interview (study) and real-time feedback to identify improvements. (IV+)	CW; HCI; QS; ICA
3 (23)	7 (20)	E11	Establishing a multidisciplinary governance committee to promote usage, track compliance, provide training and plan for post-trial sustainability; and an external data safety board to oversee safety and AI efficacy. (I+)	GOV; QS
3 (23)	6 (17)	E12	Staggered deployment across sites. (V+)	ICA
3 (23)	3 (8)	E13	A “Model Facts” sheet designed to convey relevant information about the model to clinical end users. (II)	ethics; AI; ICA; CW
3 (23)	4 (11)	E14	Improving model training for imbalanced datasets. (II)	AI
3 (23)	5 (14)	E15	A silent prospective trial conducted while existing RRT system is in place allows independent assessment of MLA performance vs existing approach. (III)	EV; ICA; DP
2 (15)	4 (11)	E16	Conducting improvement initiatives (PDSA) cycles during implementation to quickly garner and act on clinical feedback. (III+)	CW; QS; ICA
2 (15)	4 (11)	E17	Appointing clinical champions to advocate for the tool. (II+)	ICA
2 (15)	2 (5)	E18	Implementing alternative workflows during peak hours and around staff times. (IV+)	CW; ICA
2 (15)	3 (8)	E19	Teaching clinicians how to interpret risk scores. (IV+)	CW; ET; ICA
2 (15)	4 (11)	E20	Strong support from senior leadership. (I+)	ICA; GOV
2 (15)	4 (11)	E21	Increasing trust in the model as clinicians experienced the algorithm making correct predictions and detecting cases that clinicians miss. (IV+)	ICA
2 (15)	2 (5)	E22	Creating a data dictionary to harmonize data for the model across different sites/EHR systems. (V)	DP
2 (15)	4 (11)	E23	Integrating advance care planning into MLA-linked actions (palliative care built in). (III/IV)	ET; CW
2 (15)	3 (8)	E24	Incorporating the patient into care decisions; for example, developing a clinician script to explain to patients why the clinician is suddenly evaluating them. (III/IV)	ET; CW
2 (15)	4 (11)	E25	Quality tracking postimplementation and sustainability of solution. (IV/V)	EV; QS
2 (15)	2 (5)	E26	Utilizing commonly collected EHR data for the model so that the model is transferable. (II)	DP; AI

Includes the number and percentage (*n* = 13) of groups (column 1) and the number and percentage (*n* = 35) of studies (column 2) reporting each enabler. The last column contains the mapping to SALIENT components and elements. Salient components are: HCI, human-computer interface; AI, artificial intelligence model; CW, clinical workflow; DP, data pipeline. SALIENT elements are: ICA, implementation, change management, and adoption; EV, evaluation; RL, regulatory and legal; QS, quality and safety; Ethics, privacy, transparency, and equity; GOV, governance.

The most commonly reported enablers (ie, those identified by at least 5 groups) were clinician involvement throughout implementation (E01), methods identified to reduce false alarms (E02), linking the alert with clinician action (E03) and using more variables in the MLA than just vital signs (E04). Fourteen (35%) enablers were each identified in just one group, the median number of mentions per group being 5.5, with groups H and J accounting for 40% and 24% of mentions, respectively.

**Figure 3.**
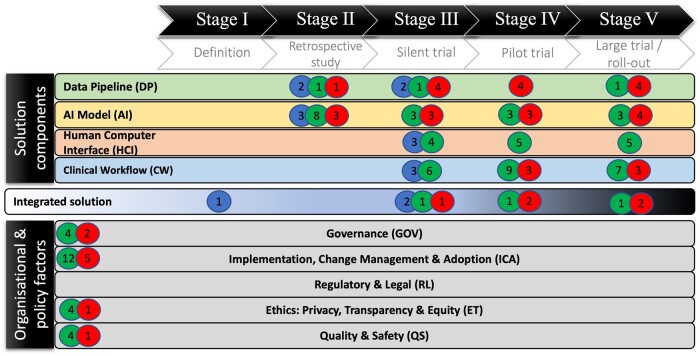
Mapping of enablers (green circles), barriers (red circles), and uncertainties (blue circles) to the SALIENT end-to-end clinical Artificial Intelligence(AI) implementation framework. The number in each circle refers to the number of that factor that is applicable at that point in the framework.

Overall, 89% of all barriers and enablers were AI task agnostic, with 2 barriers (B02, B10) and 5 enablers (E04, E15, E23, E30, E32) specific to clinical deterioration prediction. All barriers and enablers were mapped to the SALIENT AI implantation framework (see Figure 3) with most applicable (*N* = 30) to stage IV (pilot trial) and V (Roll-out) and least applicable (*N* = 13) to stage II (retrospective study). Most barriers were identified for AI (*N* = 7), data pipeline (*N* = 5) components and implementation, change and adoption (*N* = 5) element, with no barriers for the HCI component. Most enablers related to the implementation, change and adoption element (*N* = 12), and AI component (*N* = 8). Neither barriers, nor enablers were found for the regulatory and legal policy element.

#### Uncertainties

Table 6 identifies the 14 most commonly reported process uncertainties (ie, reported by >10 groups) during implementation, grouped according to differences between studies within SALIENT components, that is, outcome definition, types of MLA used, data pipelines, clinical workflows, HCIs, and implementation evaluation methods.

**Table 6.** Implementation uncertainties reported by at least 10 groups.

Group count (%)	Study count (%)	ID	Uncertainties (SALIENT stage)	SALIENT component or element
14 (100)	31 (86)	U01	What outcome basis for train/evaluate?	Definition
14 (100)	36 (100)	U02	Which Artificial Intelligence (AI) model: machine vs deep learning (II)	AI
14 (100)	30 (83)	U03	How many and which variables? (II)	AI
13 (92)	29 (80)	U04	How early to target alerts? (too early—no symptoms/signs, too late, no clinical utility). (II)	AI
14 (100)	33 (91)	U05	What data access approach to use: direct to EHR or from separate data warehouse data (II/III)	DP
13 (92)	17 (47)	U06	What level of pipeline sophistication can be supported: model performance vs engineering effort including inter-admission variables. (II/III)	DP
14 (100)	23 (63)	U07	Whether dedicated vs distributed model of alert handling (III)	CW
13 (92)	31 (86)	U08	What determines the setpoint decision. Who/how is it set? (III)	CW
13 (92)	16 (44)	U09	MLA output configuration: Binary vs continuous vs multitier (III)	CW
13 (92)	31 (86)	U10	Whether integrated within EHR or not and if not, sent via tablets/phones/pager (III)	HCI
12 (85)	19 (52)	U11	Whether individual notification (hard alert) or aggregated dashboard (soft alert) and what information is provided with the alert. (III)	HCI
10 (71)	16 (44)	U12	Alert management: Which alert timing: suppression of alerts after first alert; one time or repeat {including frequency of alert generation}. (III)	HCI
0 (0)	0 (0)	U13	Which metrics to use. (III)	EV
14 (100)	35 (97)	U14	What process to follow: Silent trial or not and which trial method. Pilot or no pilot. (II)	EV

Includes the number and percentage (*n* = 14) of groups (column 1) and the number and percentage (*n* = 36) of studies (column 2) reporting each uncertainty. The uncertainties are grouped beneath each Salient component and stage. Salient components are: HCI, human-computer interface; AI, artificial intelligence model; CW, clinical workflow; DP, data pipeline. SALIENT elements are: EV, evaluation.

##### Definition uncertainties (U01)

It remains unclear whether, and if so, how, chosen outcomes affect MLA effectiveness or clinical impact. Twenty-one different composite definitions of clinical deterioration were used with 11 individual outcomes identified (see Appendix SG, Tables SG1 and SG2). The most popular outcome measures were transfer to ICU (75%, *N* = 28), in-hospital death (61%), and cardiac arrest (36%) with each of the remaining 8 outcomes used in 3 or less studies. Specific outcome challenges included data limitations,^7^^,^^33^ inconsistencies with using ICU transfer^30^^,^^31^^,^^43^^,^^44^^,^^49^^,^^52^^,^^57^^,^^59^ and differences in how palliative cases were managed.^31^^,^^34^^,^^43^^,^^50^

##### AI model uncertainties (U02-U04)

The rationale for selecting a specific MLA (U02) included comparing different MLAs,^7^^,^^40^^,^^41^ discounting complex MLAs for lack of transparency^43^ and limiting MLAs to those supported by the group’s EHR.^33^ Half the groups (A, B, F, H, I, J, L) employed logistic regression models, of which 3 (A, F, H) were used by groups reporting decreased mortality after implementation; 1 (G) of 3 groups (E, G, N) using random forest showed similar results. Only group (D) used a deep learning model. The number of AI input variables (U03) ranged from 4 (D) to 526 (J) with a median of 43. All studies reporting decreased in-hospital mortality used less than 39 variables.^40^^,^^44^^,^^55^^,^^60^ Justification for variable selection included those commonly collected within the EHR^29^^,^^33^^,^^44^^,^^45^^,^^48^; not prone to missing or poor quality data^57^; based on prior reviews and clinician input^7^^,^^41^; purpose-built for the MLA, for example, nurse worry factor^38^; and reduced number using statistical methods such as recursive feature elimination.^6^^,^^7^^,^^29^^,^^41^^,^^43^ Targeting how early to predict deterioration (U04) involved reconciling (1) the sensitivity and PPV of the MLA; (2) the maximum time window (in number of hours) in which positive cases equaled positive alerts prior to the deterioration outcome, variously set to 12 h (H, I), 24 h (D, F, J, K, L, N), and 48 h (E); and (3) the clinical utility of the alert in providing additional time for clinicians to act in a directed way prior to clinicians suspecting deterioration but not so long before that clinicians could see no signs of deterioration and would not know how to respond.^33^^,^^51^^,^^54^

##### Data pipeline uncertainties (U05-U06)

Thinking was split (U05) over whether to use EHR data directly (A, B, D, I, K, L, N), or employ an external data warehouse (E, F, G, H, J, M) on the basis that, “existing inpatient EMRs were not designed with complex calculations in mind”^59^^(p394)^ and do not universally support real-time data streaming.^29^ Many groups reflected on trade-offs associated with data pipeline sophistication (U06). More sophisticated pipelines involving complex calculations needed to be moved out of the EHR^61^ but could also allow higher prediction refresh rates, ranging from immediate updates (A, D, I) to quarter-hourly (K), hourly (F, H, J), 2-hourly (G), and 4-hourly (E). Incorporating inter-admission data, such as comorbidities, into MLAs could also improve performance, but render the data pipeline more complex.^33^^,^^37^^,^^43^

##### Clinical workflow uncertainties (U07-U09)

Group H alone centralized alerting processes (U07) by using dedicated off-site clinical personnel to monitor alerts, minimize alert fatigue and clinical burden on Rapid Response Team (RRT) staff, and enhance standardization and clinician acceptance.^32^^,^^60^ All other groups employed decentralized alerting of-ward nursing staff. Group A switched from decentralized to a more centralized approach after establishing that alerting the charge nurse had no impact on clinical outcomes,^28^ instead redirecting alerts to the RRT nurse.^44^ The MLA alert threshold or setpoint determines the numbers of alerts and represents a trade-off between sensitivity and PPV (U08), with nearly all groups deciding this based solely on ensuring a clinically manageable workload to minimize false alarms,^32^^,^^34^^,^^37^^,^^42^^,^^44^^,^^47^^,^^53^^,^^58^ yielding 3-10 alerts/day/100 patients (see Appendix SH). These thresholds resulted in widely ranging sensitivities (25%-63%), PPV (10%-40%), and specificities (78%-98%). MLA outputs could be configured within clinical workflows (U09) as continuous index readouts (E, L), binary alerts (A, C, H, M), or multitier cut-offs (B, F, G, I, J, K) such as red-amber-green^33^^,^^55^ or high-medium-low risk levels.^38^ Justification for configuration choice was usually absent, although influenced by clinicians in 2 studies.^29^^,^^33^ Groups reporting reduced mortality used both multitier (F, G) and binary thresholds (A, H)^44^; however, group H integrated their binary threshold within a multitier rapid response system^51^ and group A was moving from a binary to multitiered threshold.^28^^,^^44^

##### Human-computer interface uncertainties (U10-U12)

Location of MLA output was split (U10) between groups integrating the outputs into the EHR (H, I, L), or displaying or sending the outputs externally (A, E, G, J, K, M) or both (B, D, F). According to Nestor et al,^50^ EHR-Integration enabled nurses to allocate staff more efficiently and clinicians to monitor patients, but potentially requiring expensive EHR changes. The delivery interface (U11) for MLA outputs also varied between groups: hard alerts via pagers or phones (A, E, G, K); soft alerts within a dashboard or screen (H, I, L, M); or both (B, D, F, J). Soft alerts were used by group H, where a dedicated nurse could constantly monitor for changes^32^^,^^47^^,^^51^ and by other groups to provide enriched information using risk-based color coding, cross-patient views, and graphical displays.^32^^,^^33^^,^^55^^,^^56^ In preventing alert fatigue, alerts were commonly suppressed across groups (D, G, H, J, K): (1) 4^39^, 8^40^, 21^53^ or 48^34^ h after the first alert; (2) within 2 h^39^ or soon after,^50^ admission; (3) if later scores varied less than 10%^40^; (4) for patients moving from the ICU^34^; (5) where the risk level did not increase^54^; and (6) for other strategic reasons based on clinician feedback.^7^^,^^32^^,^^51^

##### Evaluation uncertainties (U13, U14)

Evaluation (U13) proved challenging, with a wide range of metrics being used within and across groups, with no standardization. Only 2 groups (E, J) reported pre- and postimplementation evaluations of MLA performance using the same metrics.^30^^,^^34^ Also, not all groups conducted evaluations at all stages of implementation (U14 and refer to Appendix SI): 71% reported silent or prospective evaluations and half reported small-scale clinical trials, with 71% of the latter also conducting silent evaluations. Silent evaluations ranged from 0.5 to 10 months, average 4.4 and trials ranged from 1 to 24 months, average 10.1. All groups reporting reduced hospital mortality conducted small-scale trials (∼10 months) and 75% conducted silent evaluations (∼2.7 months).

All uncertainties were mapped to the SALIENT framework (see Figure 3) and were AI task agnostic. All but one uncertainty (U01) was relevant to SALIENT stages II (retrospective evaluation) and III (prospective evaluation), but were otherwise fairly evenly spread across the SALIENT components (AI, clinical workflow, data pipeline, and HCI) and evaluation element.

## Discussion

Our review identified 12 groups, predominantly US-based, who trialed or implemented clinical deterioration prediction MLAs within their hospital(s). Of 5 groups reporting hospital mortality, 4 saw a reduction after MLA implementation, although only statistically significant in the study by Winslow et al^55^ (group F), which also reported the most (*n* = 6) clinical process indicators with statistically significant improvements, including in median hours between alert and escalation, repeat vital signs taken and lactate orders made within 2 h. Winslow et al conducted a before-after study with a 10-month control period in which the MLA operated silently without efferent arm engagement, a 2-month implementation period, and then a 10-month intervention period. A target cohort was defined, based on high and medium risk MLA alert thresholds, which were the same for both control and intervention periods. While mortality for this target group declined significantly in the intervention period, the same was seen for the nontarget, nonalerted patient cohort, indicating possible confounder factors, such as clinician training, altered clinical workflows, and Hawthorne effects from project focus on clinical deterioration.

Other groups reporting reduced mortality were seriously confounded for the same reasons, which are difficult to control or adjust for. This problem is reflective of the dual nature of implementing MLAs or any kind of early warning system: MLAs provide the afferent arm, but achieving improvement in clinical outcomes relies on an effective medical response to the alert (efferent arm). In this sense the fidelity with which an efferent arm functions will influence or moderate the effects of the MLA. Although our longitudinal analysis attempted to identify causal steps between MLA evaluation results at each implementation stage and changes in clinical processes and ultimately in in-house mortality, ascertaining the contribution of the efferent arm to changes in outcome was not possible because of insufficient samples, poor reporting of MLA performance after the retrospective stage and different efferent arms.

Stage II (retrospective) MLA performance for groups reporting improved in-hospital mortality varied widely between 0.78 and 0.92 for AUROC, 0.49 and 0.93 for sensitivity, and 0.07 and 0.38 for PPV. Performance was rarely reported after this stage and when it was it degraded between stages II and III (prospective)^29^^,^^33^^,^^37^^,^^42^ and III and IV (trial),^34^ further challenging a convincing link between MLA performance and clinical outcomes. The highest retrospective MLA performance was reported by group K (AUC = 0.94), who reported increased in-hospital mortality, confirming that retrospective MLA performance is insufficient alone to affect positive clinical outcomes.

Older MLA technologies, such as logistic regression used in three-quarters of MLAs and using less than 39 variables, appeared sufficient for alerting purposes as they were used by the few groups reporting significantly reduced in-hospital mortality. However, as effector arms also influence outcomes, this may not constitute definitive evidence of the impact of the type of MLA or number of variables on clinical outcomes. Only one group (*n* = 12) used a deep learning model whose clinical impact was not reported.^39^

Two strategies were commonly used to combat alert fatigue: (1) nearly all groups configured their MLA alert threshold to a high level of precision sufficient to limit the number of alerts per patient per day, but at the expense of lower sensitivity, for example, Brajer et al^37^ had to reduce the sensitivity of their MLA by ∼20% to reduce alerts per day per 100 patients from 11.9 to 6; and (2) 5 groups used alert suppression after the first alert, although the suppression period varied markedly from 4 to 48 h and potential impact on clinical follow-through and care outcomes was not investigated.

Definitions of clinical deterioration outcomes are diverse, thereby preventing meaningful MLA performance comparison between groups. Eleven outcomes were identified, with 21 variants being used across groups to train and evaluate their MLAs. Transfer to ICU, the most frequently reported outcome, was particularly problematic as it is subject to different hospital admission protocols, clinician preferences and biases, and patient-level factors.^30^^,^^31^^,^^44^^,^^52^^,^^57^^,^^59^

Pilot trials (SALIENT stage IV) were employed by half the groups and 71% of the groups performed silent trials (SALIENT stage III). Silent trials were used for MLA threshold setting,^40^^,^^53^ final safety testing,^54^^,^^59^ identifying patient types reaching the threshold,^58^ finalizing response arm protocols,^61^ identifying unanticipated issues with models and data pipelines,^34^^,^^54^ and collecting feedback from users and building system trust.^34^

### Strengths and limitations

To our knowledge, this study is the first to undertake a systematic review of clinical deterioration prediction algorithms deployed or trialed in clinical settings, identify barriers, enablers, and uncertainties relevant to implementation, and map these to a single end-to-end implementation framework. Unlike similar reviews,^4^^,^^16^^,^^67–70^ we conducted a novel 2-stage literature review where, in the second stage, we identified related studies before or after the principal deployment study, thereby providing evidence across the whole MLA implementation process. We also found the findings of each study could be mapped to one or more stages within the SALIENT implementation framework, thereby making explicit when and where these factors arise within the multistage implementation process. This novel approach helps close the gaps in current implementation guidance and offers a pragmatic overview for use by clinicians, informatics personnel, and managers engaged in AI implementation planning.

Limitations relate to the small number of empirical studies of deployed algorithms, heterogeneity of performance reporting, underreporting of postimplementation performance metrics, and potential publication bias. Although RoB for mortality reporting studies was moderate to high, 4 of 5 groups reported reductions in mortality, with one being statistically significant, underscoring the need to further evaluate this relationship in future work. Our study is limited by the scope of SALIENT. It does not include the full AI lifecycle, for example AI decommissioning and maintenance, and may be missing other pragmatic elements, such as might be found in stakeholder-based models.^71^

## Conclusions

Implementing MLAs within adult hospital care settings to predict clinical deterioration can potentially change clinical practice and improve mortality. However, insufficient number of cases, moderate-high levels of bias, and lack of uniform MLA performance reporting across implementation stages prevents establishment of a causal link. Enablers and barriers to successful MLA implementation have been identified, in particular strategies for combatting alert fatigue, and the value of conducting both silent and live pilot trials. Noteworthy too was the finding that older and simpler logistic regression MLAs appeared sufficient to achieve acceptable levels of performance and enable clinical impact.

However, multiple implementation uncertainties throughout the multistage process require further research to quantify effect, with likely more yet to be identified as MLAs and their implementation evolve. Use of the SALIENT end-to-end implementation framework helps identify exactly where in the implementation pipeline these barriers, enablers, and uncertainties are located, providing a practical roadmap for stakeholders wishing to implement clinical deterioration prediction algorithms.

## Supplementary Material

ocad220_Supplementary_Data

## Data Availability

There are no new data associated with this article.
