# Supplementary material for: Systematic review and longitudinal analysis of implementing Artificial Intelligence to predict clinical deterioration in adult hospitals: what is known and what remains uncertain
Source: J Am Med Inform Assoc. 2023 Nov 14;31(2):509–24. doi: 10.1093/jamia/ocad220 (PMC10797271; doi:10.1093/jamia/ocad220)
Supplement: ocad220_Supplementary_Data [file ocad220_supplementary_data.docx]

Supplementary Appendices

Table of Contents

[Appendix A: Search details 3](#_Toc149202003)

[Pubmed search details 3](#_Toc149202004)

[Embase search details 4](#_Toc149202005)

[Scopus search details 5](#_Toc149202006)

[Web of Science search details 6](#_Toc149202007)

[CINAHL search details 9](#_Toc149202008)

[Appendix B. Selection criteria 9](#_Toc149202009)

[Appendix C. Extracted data items 11](#_Toc149202010)

[Paper Information 11](#_Toc149202011)

[Primary algorithm information 11](#_Toc149202012)

[Primary data pipeline information 11](#_Toc149202013)

[Retrospective dataset details (duplicate for external validation) 11](#_Toc149202014)

[Silent trial dataset details 12](#_Toc149202015)

[Pilot/small & large trial or roll-out details 12](#_Toc149202016)

[Evaluation, barriers, enablers and uncertainty points 12](#_Toc149202017)

[Appendix D: Risk of Bias Assessment 13](#_Toc149202018)

[Appendix E: Extracted Evaluation Metrics 14](#_Toc149202019)

[Table E1: Algorithm performance metrics 14](#_Toc149202020)

[Table E2: Alert metrics 15](#_Toc149202021)

[Table E3: Patient Outcome metrics 16](#_Toc149202022)

[Table E4: Clinical process metrics 17](#_Toc149202023)

[Appendix F: Barrier and Enabler Raw Data 19](#_Toc149202024)

[Appendix G: Outcomes employed by each study 23](#_Toc149202025)

[Table G1: Criteria utilised by papers 23](#_Toc149202026)

[Table G2: Clinical deterioration outcome used by each paper 23](#_Toc149202027)

[Appendix H: Algorithm setpoint decisions 24](#_Toc149202028)

[Table H1: Alert threshold setting basis 24](#_Toc149202029)

[Appendix I: Evaluation Stages by Group 26](#_Toc149202030)

[Table I1: Evaluation Stages (SALIENT stage) by Group 26](#_Toc149202031)

# Appendix A: Search details

## Pubmed search details

| Search number | Query | Results |
| --- | --- | --- |
| 12 | #4 AND #5 AND #7 AND #8 NOT #11 | 206 |
| 11 | #9 NOT #10 | 2,712,818 |
| 10 | (man[Title/Abstract]) OR (men[Title/Abstract]) OR (woman[Title/Abstract]) OR (women[Title/Abstract]) OR (adult[Title/Abstract]) | 2,274,449 |
| 9 | ("Child"[Mesh]) or ("Infant"[Mesh]) or ("Adolescent"[Mesh]) or (baby[Title/Abstract]) OR (child*[Title/Abstract]) OR (infant*[Title/Abstract]) or (boy*[Title/Abstract]) or (girl*[Title/Abstract]) or (teenager[Title/Abstract]) or (youth[Title/Abstract]) or (pediatr*[Title/Abstract]) or (paediat*[Title/Abstract]) OR (adolescent[Title/Abstract]) | 3,206,429 |
| 8 | ("clinical deterioration"[MeSH Terms]) OR (clinical deteriorat*[Title/Abstract]) OR (patient deteriorat*[Title/Abstract]) or ("early warning score*"[Title/Abstract]) OR ("early warning system*"[Title/Abstract]) | 9,866 |
| 7 | ("Clinical Studies as Topic"[Mesh]) OR ("Prospective Studies"[Mesh]) OR (trial*[Title/Abstract]) OR ("clinical study"[Title/Abstract]) OR (prospective stud*[Title/Abstract]) OR ("pilot projects"[MeSH Terms]) OR (pilot stud*[Title/Abstract]) OR ("controlled before-after studies"[MeSH Terms]) OR (controlled before after stud*[Title/Abstract]) OR (roll-out[Title/Abstract]) OR (RCT[Title/Abstract]) OR (controlled trial*[Title/Abstract]) OR (non random*[Title/Abstract]) OR (prospective validation[Title/Abstract]) OR (implement*[Title/Abstract]) OR (deploy*[Title/Abstract]) OR (adopt*[Title/Abstract]) | 2,786,339 |
| 5 | predict*[Title/Abstract] OR (detect*[Title/Abstract]) OR (identif*[Title/Abstract]) | 6,593,276 |
| 4 | ("Artificial Intelligence"[Mesh]) OR (algorithm*[Title/Abstract]) OR "machine learning"[Title/Abstract] OR "prediction model*"[Title/Abstract] OR "neural network*"[Title/Abstract] OR "deep learning"[Title/Abstract] OR "Artificial Intelligence"[Title/Abstract] OR AI[Title/Abstract] OR "decision tree*"[Title/Abstract] OR "computational intelligence"[Title/Abstract] OR "machine intelligence"[Title/Abstract] OR "big data"[Title/Abstract] OR "bayesian"[Title/Abstract] OR "naive bayes"[Title/Abstract] OR "k nearest neighbour"[Title/Abstract] OR "decision support"[Title/Abstract] OR "random forest"[Title/Abstract] OR "support vector machine"[Title/Abstract] OR SVM[Title/Abstract] OR Xgboost[Title/Abstract] OR adaboost[Title/Abstract] OR "gradient boosting machine*"[Title/Abstract] OR "regression tree*"[Title/Abstract] OR "least squares"[Title/Abstract] OR "stepwise regression"[Title/Abstract] OR LSTM[Title/Abstract] OR RNN[Title/Abstract] | 534,291 |

## Embase search details

| **No.** | **Query** | **Results** |
| --- | --- | --- |
| #8 | #7 AND (2010:py OR 2011:py OR 2012:py OR 2013:py OR 2014:py OR 2015:py OR 2016:py OR 2017:py OR 2018:py OR 2019:py OR 2020:py OR 2021:py OR 2022:py OR 2023:py) | 261 |
| #7 | #6 NOT #5 | 274 |
| #6 | #1 AND #2 AND #3 AND #4 | 325 |
| #5 | (adolescen*:ti,ab,kw OR child*:ti,ab,kw OR schoolchild*:ti,ab,kw OR infant*:ti,ab,kw OR girl*:ti,ab,kw OR boy*:ti,ab,kw OR teen*:ti,ab,kw OR youth*:ti,ab,kw OR pediatr*:ti,ab,kw OR paediatr*:ti,ab,kw OR puber*:ti,ab,kw OR baby:ti,ab,kw) NOT (adult*:ti,ab,kw OR man:ti,ab,kw OR men:ti,ab,kw OR woman:ti,ab,kw OR women:ti,ab,kw) | 2534787 |
| #4 | 'patient deterior*':ti,ab,kw OR 'clinical deterior*':ti,ab,kw OR 'early warning system*':ti,ab,kw OR 'early warning score*':ti,ab,kw | 16661 |
| #3 | trial*:ti,ab,kw OR 'clinical study':ti,ab,kw OR 'prospective stud*':ti,ab,kw OR 'pilot study':ti,ab,kw OR 'controlled before-after stud*':ti,ab,kw OR 'roll-out':ti,ab,kw OR 'random controlled trial*':ti,ab,kw OR rct:ti,ab,kw OR 'controlled trial':ti,ab,kw OR 'non-random* trial':ti,ab,kw OR prospective:ti,ab,kw OR 'prospective validation':ti,ab,kw OR implement*:ti,ab,kw OR 'pre-post implementation':ti,ab,kw OR deploy*,ab,kw OR adopt*,ab,kw | 3603595 |
| #2 | predict*:ti,ab,kw OR detect*:ti,ab,kw OR identif*:ti,ab,kw | 9596099 |
| #1 | 'machine learning':ti,ab,kw OR 'prediction model*':ti,ab,kw OR 'neural network*':ti,ab,kw OR 'deep learning':ti,ab,kw OR 'artificial intelligence':ti,ab,kw OR ai:ti,ab,kw OR 'decision tree*':ti,ab,kw OR 'computational intelligence':ti,ab,kw OR 'machine intelligence':ti,ab,kw OR 'algorithm*':ti,ab,kw OR 'big data':ti,ab,kw OR bayesian:ti,ab,kw OR 'na‚àö√∏ve bayes':ti,ab,kw OR 'k-nearest neighbour':ti,ab,kw OR 'decision support':ti,ab,kw OR 'random forest':ti,ab,kw OR 'support vector machine':ti,ab,kw OR svm:ti,ab,kw OR xgboost:ti,ab,kw OR adaboost:ti,ab,kw OR 'gradient boosting machine*':ti,ab,kw OR 'regression tree*':ti,ab,kw OR 'least squares':ti,ab,kw OR 'stepwise regression':ti,ab,kw OR lstm:ti,ab,kw OR rnn:ti,ab,kw | 836221 |

## Scopus search details


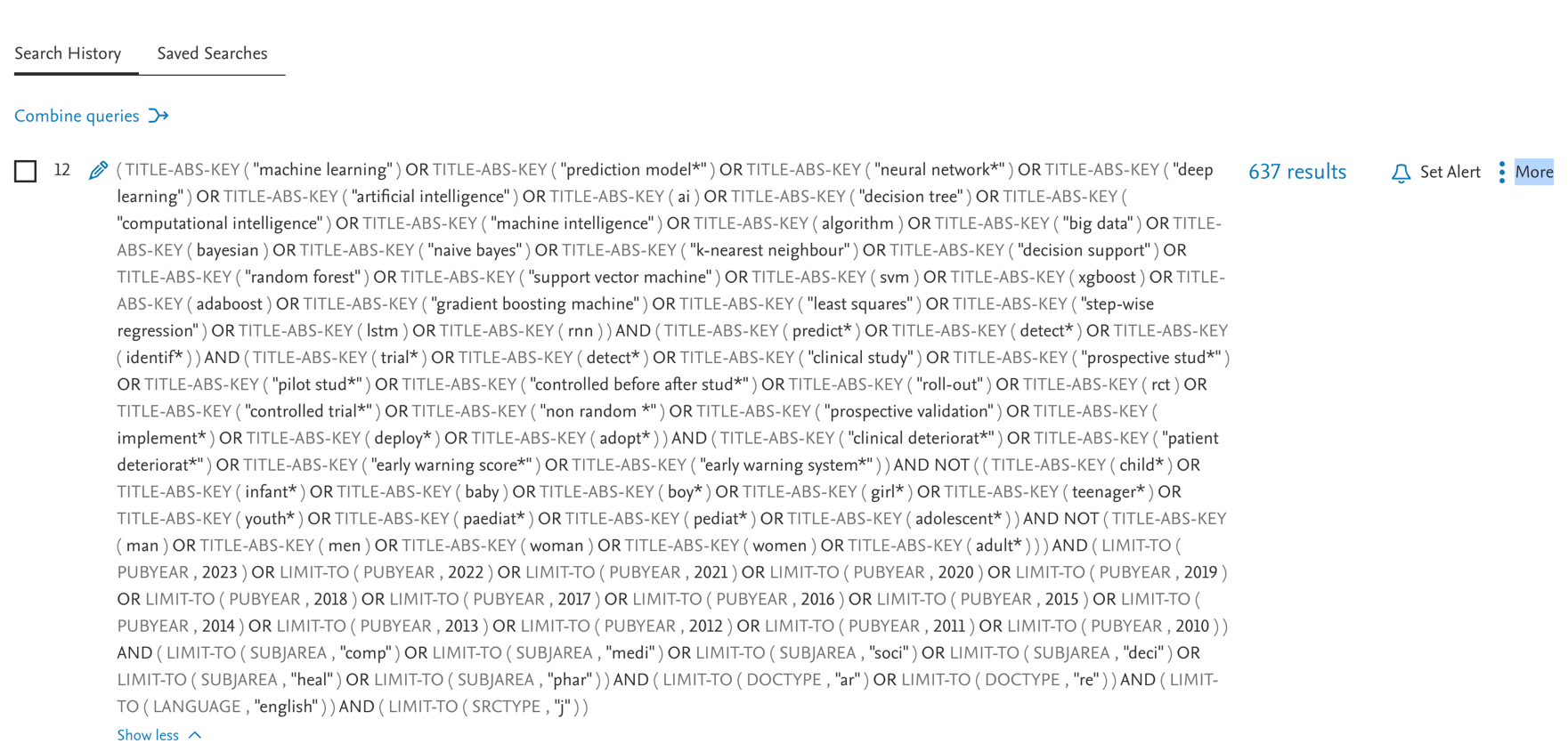

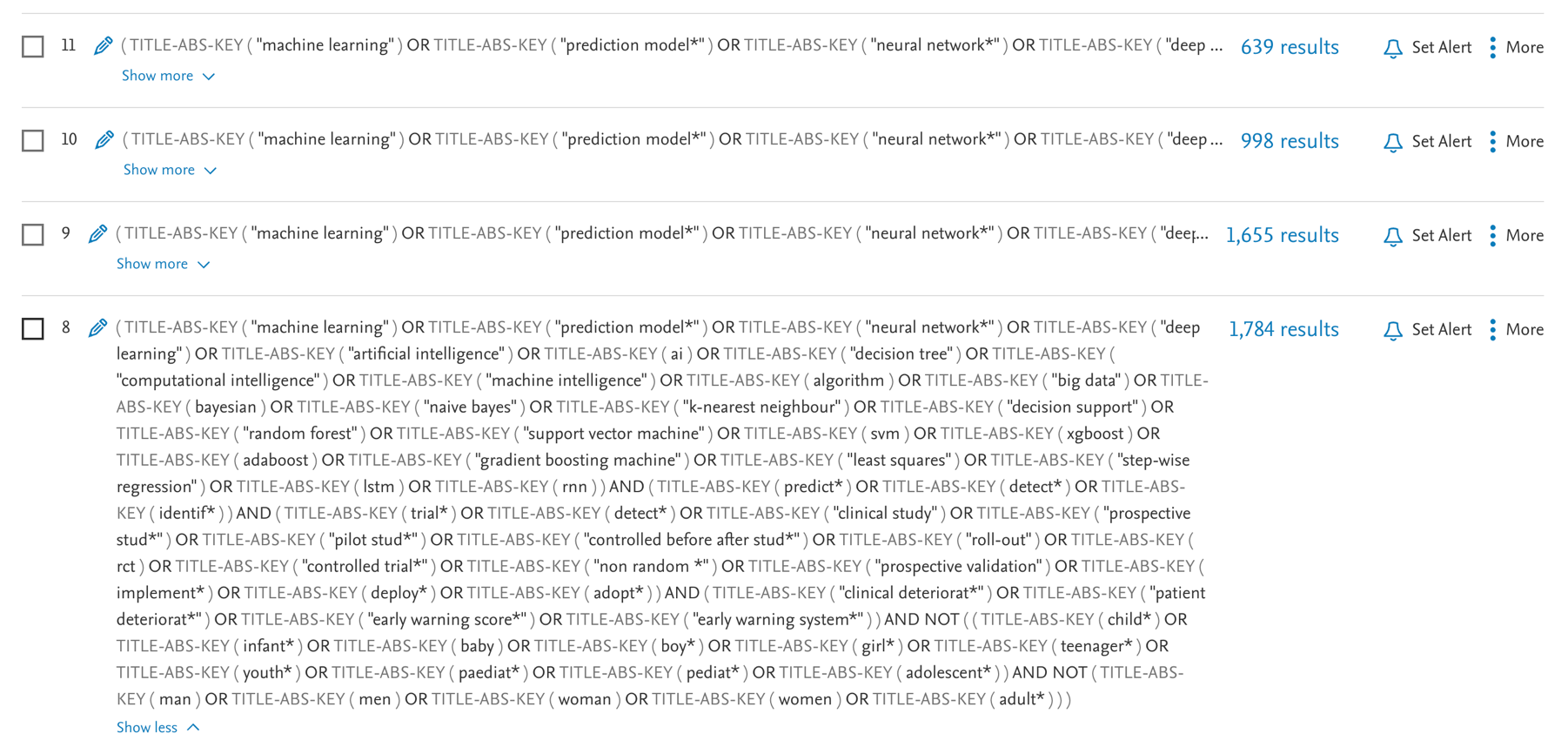


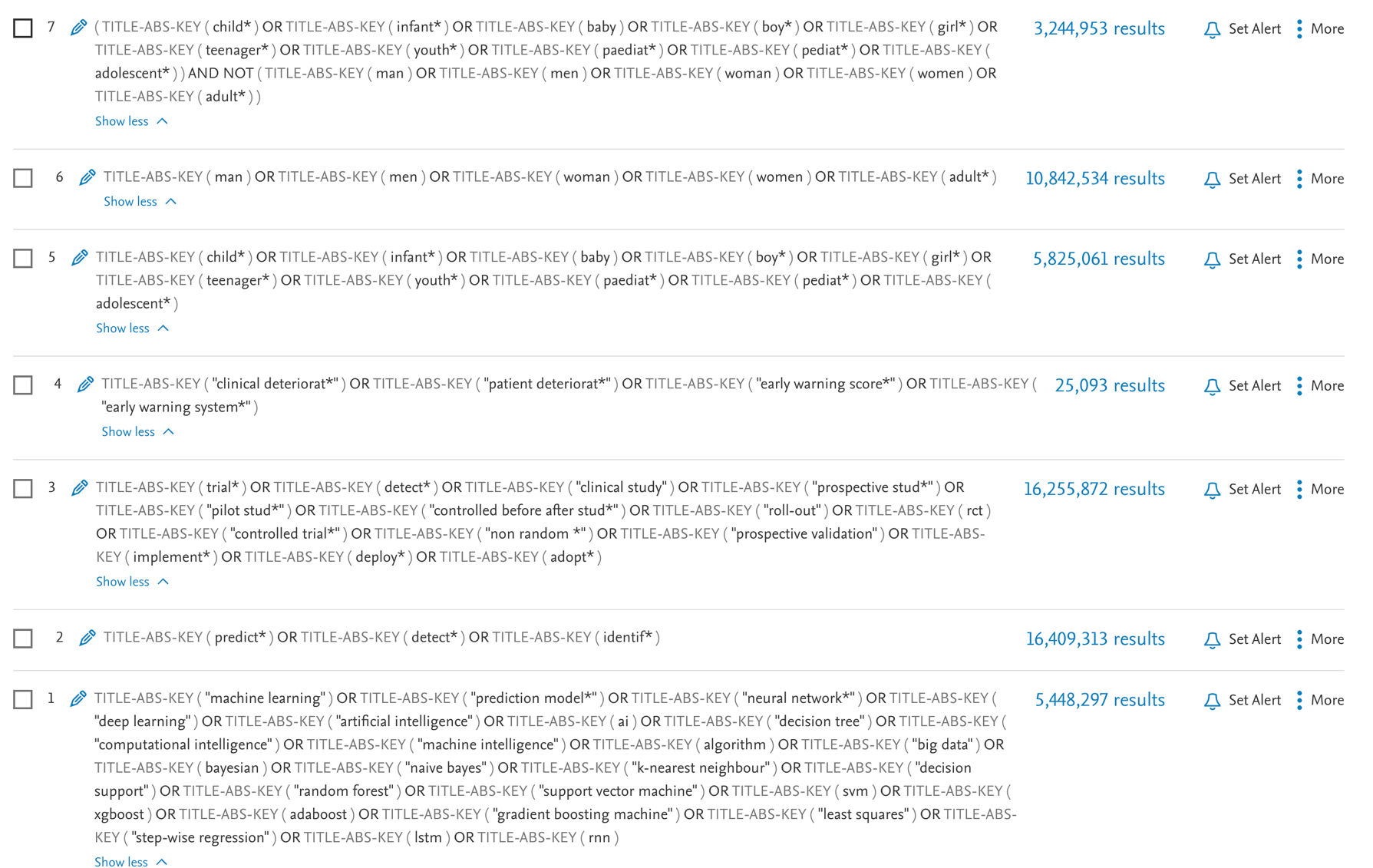


Figure A1: Scopus screenshot of final query numbers.

## Web of Science search details

| **Type** | **Search Query** | **Results** |
| --- | --- | --- |
| #10 | #4 AND #3 AND #2 AND #1 NOT #5 and 2022 or 2021 or 2020 or 2019 or 2018 or 2017 or 2016 or 2015 or 2014 or 2013 or 2012 or 2011 or 2010 (Publication Years) and Article or Review Article (Document Types) and Health Care Sciences Services or Medical Informatics or Computer Science Information Systems or Critical Care Medicine or Computer Science Interdisciplinary Applications or Computer Science Artificial Intelligence or Medicine General Internal or Respiratory System or Neurosciences or Tropical Medicine or Gerontology or Physiology or Surgery or Toxicology (Web of Science Categories) | 158 |
| #9 | #4 AND #3 AND #2 AND #1 NOT #5 and 2022 or 2021 or 2020 or 2019 or 2018 or 2017 or 2016 or 2015 or 2014 or 2013 or 2012 or 2011 or 2010 (Publication Years) and Article or Review Article (Document Types) | 418 |
| #8 | #4 AND #3 AND #2 AND #1 NOT #5 and 2022 or 2021 or 2020 or 2019 or 2018 or 2017 or 2016 or 2015 or 2014 or 2013 or 2012 or 2011 or 2010 (Publication Years) | 504 |
| #7 | #4 AND #3 AND #2 AND #1 NOT #5 | 542 |
| #6 | #5 AND #4 AND #3 AND #2 AND #1 | 23 |
| #5 | ((TI=(baby OR adolescen* OR child* OR schoolchild* OR infant* OR girl* OR boy* OR teen*OR youth* OR pediatr* OR paediatr* OR puber*)) OR (AB=(baby OR adolescen* OR child* OR schoolchild* OR infant* OR girl* OR boy* OR teen*OR youth* OR pediatr* OR paediatr* OR puber*)) OR (AK=(baby OR adolescen* OR child* OR schoolchild* OR infant* OR girl* OR boy* OR teen* OR youth* OR pediatr* OR paediatr* OR puber*))) NOT ((TI=(adult* OR man OR men OR woman OR women)) OR (AB=(adult* OR man OR men OR woman OR women)) OR (AK=(adult* OR man OR men OR woman OR women))) | 2514836 |
| #4 | (TI=("patient deterior*" OR "clinical deterior*" OR "early warning system*" OR "early warning score*" )) OR (AB=("patient deterior*" OR "clinical deterior*" OR "early warning system*" OR "early warning score*" )) OR (AK=("patient deterior*" OR "clinical deterior*" OR "early warning system*" OR "early warning score*" )) | 16498 |
| #3 | (TI=(implement* OR adopt* OR deploy* OR trial* OR "clinical study" OR "prospective stud*" OR "pilot stud*" OR "controlled before-after stud*" OR "roll-out" OR "random controlled trial*" OR rct OR "controlled trial" OR "non-random* trial" OR prospective OR "prospective validation" OR implementation OR "pre-post implementation")) OR (AB=(implement* OR adopt* OR deploy* OR trial* OR "clinical study" OR "prospective stud*" OR "pilot stud*" OR "controlled before-after stud*" OR "roll-out" OR "random controlled trial*" OR rct OR "controlled trial" OR "non-random* trial" OR prospective OR "prospective validation" OR implementation OR "pre-post implementation")) OR (AK=(implement* OR adopt* OR deploy* OR trial* OR "clinical study" OR "prospective stud*" OR "pilot stud*" OR "controlled before-after stud*" OR "roll-out" OR "random controlled trial*" OR rct OR "controlled trial" OR "non-random* trial" OR prospective OR "prospective validation" OR implementation OR "pre-post implementation")) | 5719436 |
| #2 | (TI=(predict* OR detect* OR identif* )) OR (AB=(predict* OR detect* OR identif* )) OR (AK=(predict* OR detect* OR identif* )) | 12650819 |
| #1 | (TI=("machine learning" OR "prediction model*" OR "neural network*" OR "deep learning" OR "artificial intelligence" OR ai OR "decision tree*" OR "computational intelligence" OR "machine intelligence" OR "algorithm*" OR "big data" OR bayesian OR "naïve bayes" OR "k-nearest neighbour" OR "decision support" OR "random forest" OR "support vector machine" OR svmOR xgboost OR adaboost OR "gradient boosting machine*" OR" regression tree*" OR "least squares" OR "stepwise regression" OR lstm OR rnn)) or (AB=("machine learning" OR "prediction model*" OR "neural network*" OR "deep learning" OR "artificial intelligence" OR ai OR "decision tree*" OR "computational intelligence" OR "machine intelligence" OR "algorithm*" OR "big data" OR bayesian OR "naïve bayes" OR "k-nearest neighbour" OR "decision support" OR "random forest" OR "support vector machine" OR svm OR xgboost OR adaboost OR "gradient boosting machine*" OR "regression tree*" OR "least squares" OR "stepwise regression" OR lstm OR rnn)) OR (AK=("machine learning" OR "prediction model*" OR "neural network*" OR "deep learning" OR "artificial intelligence" OR ai OR "decision tree*" OR "computational intelligence" OR "machine intelligence" OR "algorithm*" OR "big data" OR bayesian OR "naïve bayes" OR "k-nearest neighbour" OR "decision support" OR "random forest" OR "support vector machine" OR svm OR xgboost OR adaboost OR "gradient boosting machine*" OR "regression tree*" OR "least squares" OR "stepwise regression" OR lstm OR rnn)) | 3545926 |

## CINAHL search details

| **#** | **Query** | **Results** |  |
| --- | --- | --- | --- |
| S10 | S8 NOT S7 | 75 |  |
|  |  |  |  |
|  |  |  |  |
| S9 | S8 NOT S7 | 79 |  |
|  |  |  |  |
|  |  |  |  |
| S8 | S1 AND S2 AND S3 AND S4 | 89 |  |
|  |  |  |  |
|  |  |  |  |
| S7 | S5 NOT S6 | 652,966 |  |
|  |  |  |  |
|  |  |  |  |
| S6 | TI adult or TI man or TI men or TI woman or TI women or AB adult or AB man or AB men or AB woman or AB women | 987,410 |  |
|  |  |  |  |
|  |  |  |  |
| S5 | TI adolescen* or TI child or TI schoolchid or TI infant or TI baby or TI girl or TI boy or TI paediatr* or TI teenagers or TI young adults or TI teen or TI youth or AB adolescen* or AB child or AB schoolchid or AB infant or AB baby or AB girl or AB boy or AB paediatr* or AB teenagers or AB young adults or AB teen or AB youth | 828,685 |  |
|  |  |  |  |
|  |  |  |  |
| S4 | TI patient deterior* or TI clinical deterior* or TI early warning | 2,074 |  |
|  |  |  |  |
|  |  |  |  |
| S3 | TI *trial* or TI study or TI prospect* or TI implement* or TI deploy* or TI adopt* or TI random or TI rct or AB *trial* or AB study or AB prospect* or AB implement* or AB deploy* or AB adopt* or AB random or AB rct | 2,818,269 |  |
|  |  |  |  |
|  |  |  |  |
| S2 | TI predict* OR AB predict* OR TI detect* OR AB detect* OR TI identif* OR AB identif* | 1,330,124 |  |
|  |  |  |  |
|  |  |  |  |
| S1 | TI machine learning or TI artificial intelligence or TI deep learning or TI neural network or TI ai or TI big data or TI prediction model or TI algorithm or TI decision tree or TI computational intelligence or TI machine intelligence or TI bayesian or TI decision support or TI naive bayes or TI k-nearest neighbour or TI svm or TI support vector machine or TI regression tree or TI least squares or TI stepwise regression or TI random forest or TI lstm or TI rnn or AB machine learning or AB artificial intelligence or AB deep learning or AB neural network or AB ai or AB big data or AB prediction model or AB algorithm or AB decision tree or AB computational intelligence or AB machine intelligence or AB bayesian or AB decision support or AB naive bayes or AB k-nearest neighbour or AB svm or AB support vector machine or AB regression tree or AB least squares or AB stepwise regression or AB random forest or AB lstm or AB rnn | 124,352 |  |
|  |  |  |  |
|  |  |  |  |

# Appendix B. Selection criteria


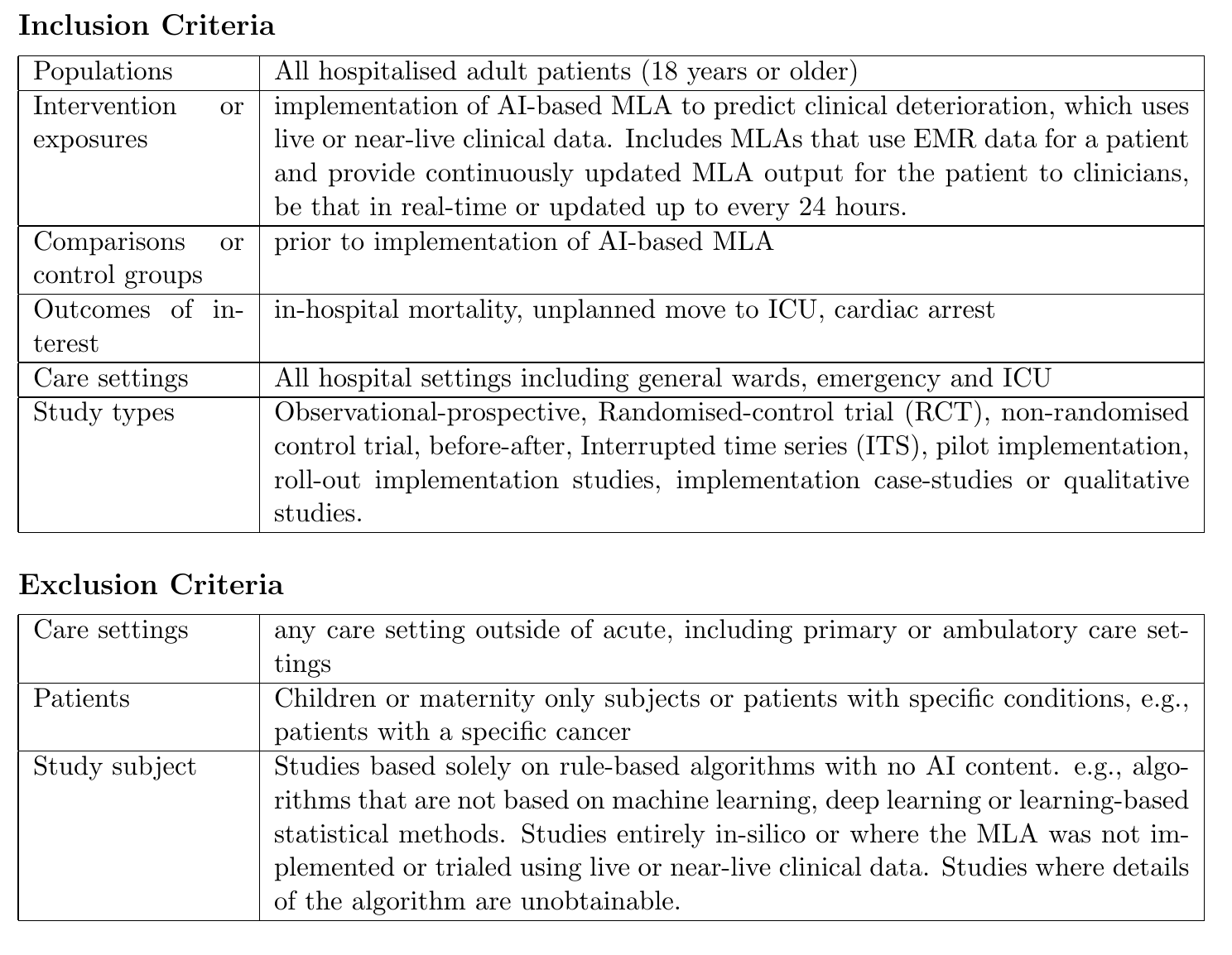


# Appendix C. Extracted data items

## Paper Information

| Year | Journal | Country/research group |
| --- | --- | --- |
| MLA name | Conflicts reported | Study objective |
| Primary location [of trial] | Was continuous monitoring involved [y\|n] | Reported study limitations |
| Was it a clinical trial [y\|n] | Outcomes used | Outcome detail |
| Outcome timing |  |  |

## Primary algorithm information

| Type | Number of variables used | Same as study [reference to another study] |
| --- | --- | --- |
| Based on study [reference to another study] | Special notes on algorithm | Output [binary\|score] |

## Primary data pipeline information

| Data collection frequency | Identify outliers [y\|n] | Method of handling outliers |
| --- | --- | --- |
| Normalization method | Bucketing method | Data sliding window employed |
| Bucket aggregation method | Data sample description | Bucket imputation method |
| Imputation method if no previous value | Score/alert recalculation timing | Other pipeline notes |

## Retrospective dataset details (duplicate for external validation)

| Location (if different) | Study type | Dates |
| --- | --- | --- |
| Months of data | Number of visits | Number of patients |
| Number of samples | Outcome rates | Omissions/filters applied to data |
| Prediction window labeling | Training data used | Training data omitted |
| Unclear about training [y\|n] | Train/test split method | MLA cutoff decision |
| Model used | Variables used | Variable importance done? [y\|n] |

## Silent trial dataset details

| Location (if different) | Study type | Dates |
| --- | --- | --- |
| Months of data | Number of visits | Number of patients |
| Number of samples | Outcome rates | Omissions/filters applied to data |
| MLA cutoff decision | Silent trial description | Alert timing |

## Pilot/small & large trial or roll-out details

| Location (if different) | Study type | Dates |
| --- | --- | --- |
| Months of data | Model used | Model changes, settings |
| Trial randomization/control & intervention details | Control group details | Intervention group details |
| Special training/education | Data omission | Control vs intervention group differences |
| Number of control patients | Number of intervention patients | Outcome rates |
| Alert frequency | Where did alerts go | Instructions for what to do with alerts |
| Timing of alerts | Summary outcome | Post-study actions |

## Evaluation, barriers, enablers and uncertainty points

| Evaluation stage | Unit of performance testing (i.e., number of admissions or patients or samples) | Specified cut-off |
| --- | --- | --- |
| Hours before event | Metric used | Metric value (or ‘graph’ if graph used) |
| Barriers | Enablers | Uncertainty points |

# Appendix D: Risk of Bias Assessment

|  |  | **Confounding** | **Selection of participants (Randomization for RCT)** | **Classification of interventions** | **Deviation from intended interventions** | **Missing data** | **Measurement of outcomes** | **Selection of reported results** | **Overall** |
| --- | --- | --- | --- | --- | --- | --- | --- | --- | --- |
| **Group A ()** | |  |  |  |  |  |  |  |  |
|  | Bailey et al., 2013 |  | Low |  | Some | Some | Low | Low | Some |
|  | Kollef et al., 2014 |  | Low |  | Some | Some | Low | Low | Some |
| **Group F (eCart)** | |  |  |  |  |  |  |  |  |
|  | Winslow et al., 2022 | Moderate | Low | Low | Moderate | Low | Low | Low | Moderate |
| **Group G (MEWS++)** | |  |  |  |  |  |  |  |  |
|  | Levin et al., 2022 | Moderate | Serious | Low | Moderate | Moderate | Serious | Moderate | Serious |
| **Group H (AAM)** | |  |  |  |  |  |  |  |  |
|  | Escobar et al., 2020 | Moderate | Low | Low | Low | Low | Low | Low | Moderate |
| **Group K (MC-EWS)** | |  |  |  |  |  |  |  |  |
|  | Romero-Brufau et al., 2021 | Serious | Low | Low | Low | Low | Moderate | Serious | Serious |
|  |  | | | | |  |  |  |  |

**Table D1: Risk of bias (ROB) assessment of studies reporting mortality**

All studies are assessed using ROBINS-I tool^24^ for non-randomised studies, except for Kollef et al. and Bailey et al., which are assessed using the Cochrane Risk-of-bias-2 tool for randomized studies.^25^ The bias scale is: Low risk of bias (the study is comparable to a well-performed randomized trial with regard to this domain); Moderate risk of bias (the study is sound for a non-randomized study with regard to this domain but cannot be considered comparable to a well-performed randomized trial); Serious risk of bias (the study has some important problems); Critical risk of bias (the study is too problematic to provide any useful evidence on the effects of intervention).

# Appendix E: Extracted Evaluation Metrics

For each table in this section: The group count, column (2), specifies the number of groups that use the metric and group coverage, column (3), reports the percentage of all groups (n=15) that use the metric. The paper count, column (4), specifies the number of papers that report the metric and the paper coverage, column (5), specifies the percentage of all papers that report any metrics (n=33) that report this metric.

## Table E1: Algorithm performance metrics

| **Metric Number** | **Group count** | **Group coverage** | **Paper count** | **Paper coverage** | **metric** |
| --- | --- | --- | --- | --- | --- |
| 1 | 14 | 100% | 27 | 82% | Sensitivity |
| 2 | 14 | 100% | 25 | 76% | C-statistic / area under receiver operating curve, AUROC |
| 3 | 12 | 86% | 19 | 58% | Positive predictive value, PPV |
| 4 | 11 | 79% | 21 | 64% | Specificity |
| 5 | 6 | 43% | 10 | 30% | Negative predictive value, PPV |
| 6 | 4 | 29% | 5 | 15% | Mean score of event cohort by hours before event |
| 7 | 3 | 21% | 5 | 15% | Area under precision recall curve (AUPRC) |
| 8 | 3 | 21% | 5 | 15% | Number needed to treat/examine (NNE) |
| 9 | 3 | 21% | 3 | 9% | Precision (PPV) recall plot |
| 10 | 3 | 21% | 3 | 9% | Calibration plot |
| 11 | 2 | 14% | 4 | 12% | Net reclassification index, NRI |
| 12 | 2 | 14% | 4 | 12% | Accuracy |
| 13 | 2 | 14% | 4 | 12% | Cumulative percent of event cohort detected by system by hours before event |
| 14 | 2 | 14% | 3 | 9% | F-measure |
| 15 | 2 | 14% | 3 | 9% | Cumulative percent of cohorts (sensitivity) (event/non-event) by risk score of MLA |
| 16 | 2 | 14% | 3 | 9% | PPV by risk score of MLA |
| 17 | 2 | 14% | 2 | 6% | Work up detection ratio |
| 18 | 2 | 14% | 2 | 6% | False positive rate |
| 19 | 2 | 14% | 2 | 6% | NNE vs sensitivity plot |
| 20 | 2 | 14% | 2 | 6% | Sensitivity at each hour before positive event |
| 21 | 1 | 7% | 2 | 6% | Specificity by risk score of MLA |
| 22 | 1 | 7% | 2 | 6% | NPV by risk score of MLA |
| 23 | 1 | 7% | 1 | 3% | Positive liklihood ratio |
| 24 | 1 | 7% | 1 | 3% | Negative liklihood ratio |
| 25 | 1 | 7% | 1 | 3% | False negative rate |
| 26 | 1 | 7% | 1 | 3% | AUC by time after admission |
| 27 | 1 | 7% | 1 | 3% | Percent change in sensitivity by risk score of MLA |
| 28 | 1 | 7% | 1 | 3% | Percent change in specificity by risk score of MLA |
| 29 | 1 | 7% | 1 | 3% | Percent change in PPV by risk score of MLA |
| 30 | 1 | 7% | 1 | 3% | Percent change in NPV by risk score of MLA |
| 31 | 1 | 7% | 1 | 3% | Percent of event detected compared with standard practice (e.g., RRT) |
| 32 | 1 | 7% | 1 | 3% | (1-specificity) at each hour before positive event |
| 33 | 1 | 7% | 1 | 3% | Sensitivity versus probability of an adverse event |

Table E1: Algorithm performance metrics sorted in descending order of group coverage.

## Table E2: Alert metrics

| **Metric Number** | **Group count** | **Group coverage** | **Paper count** | **Paper coverage** | **metric** |
| --- | --- | --- | --- | --- | --- |
| 1 | 7 | 50% | 10 | 30% | Median alert hours before event |
| 2 | 3 | 21% | 4 | 12% | Average alert hours before event (range) |
| 3 | 2 | 14% | 2 | 6% | MACD: Mean alarm count per day |
| 4 | 2 | 14% | 2 | 6% | Percent of patients escalated to ICU within 12hrs of alert |
| 5 | 2 | 14% | 2 | 6% | Percent of patients escalated to ICU within 24hrs of alert |
| 6 | 2 | 14% | 2 | 6% | Sensitivity vs alerts per day |
| 7 | 1 | 7% | 2 | 6% | MACPD vs sensitivity plot |
| 8 | 1 | 7% | 1 | 3% | Alerts per [hospital nursing] unit per day |
| 9 | 1 | 7% | 1 | 3% | Alerts per day per 10 patients |
| 10 | 1 | 7% | 1 | 3% | Mean alert count per day per mean patients per day |
| 11 | 1 | 7% | 1 | 3% | MACPD: Mean alarm count per day per 1000 beds |
| 12 | 1 | 7% | 1 | 3% | MACHP: Mean alarm count per hour per patient |
| 13 | 1 | 7% | 1 | 3% | Mean alarm count per 1000 discharges |
| 14 | 1 | 7% | 1 | 3% | Alerts per day per 10 patients by sensitivity |
| 15 | 1 | 7% | 1 | 3% | Mean number of alerts per alerted patients |
| 16 | 1 | 7% | 1 | 3% | Mean alerts per admission |
| 17 | 1 | 7% | 1 | 3% | Number of alerts by the number of hours before the alert |
| 18 | 1 | 7% | 1 | 3% | Risk of event for patients meeting alert versus those who don't |
| 19 | 1 | 7% | 1 | 3% | Association between median LOS and positive alert |
| 20 | 1 | 7% | 1 | 3% | Specificity vs alerts per day |

Table E2: Alert performance metrics sorted in descending order of group coverage.

## Table E3: Patient Outcome metrics

| **Metric Number** | **Group count** | **Group coverage** | **Paper count** | **Paper coverage** | **metric** |
| --- | --- | --- | --- | --- | --- |
| 1 | 5 | 36% | 6 | 18% | All-cause hospital mortality |
| 2 | 3 | 21% | 4 | 12% | Hospital length of stay (LOS) |
| 3 | 2 | 14% | 2 | 6% | Intensive care unit (ICU) length of stay (LOS) |
| 4 | 2 | 14% | 2 | 6% | 30-day mortality |
| 5 | 1 | 7% | 1 | 3% | Adjusted odds ratio for death |
| 6 | 1 | 7% | 1 | 3% | Interrupted time series analysis of mortality |
| 7 | 1 | 7% | 1 | 3% | Hospital LOS (survivors) |
| 8 | 1 | 7% | 1 | 3% | 30-day readmission |
| 9 | 1 | 7% | 1 | 3% | 180-day readmission |
| 10 | 1 | 7% | 1 | 3% | Death within 30 days of first alert |
| 11 | 1 | 7% | 1 | 3% | Combined in-hospital & 30-day mortality |
| 12 | 1 | 7% | 1 | 3% | Adjusted relative risk for death within 30 days after alert |
| 13 | 1 | 7% | 1 | 3% | Adjusted relative risk for favourable status at 30 days after alert |
| 14 | 1 | 7% | 1 | 3% | Hazard rate ratio for hospital discharge |
| 15 | 1 | 7% | 1 | 3% | Hazard rate ratio for survival |
| 16 | 1 | 7% | 1 | 3% | Mortality hazard ratio (high vs low risk category) for death within 20 days after alert |
| 17 | 1 | 7% | 1 | 3% | Survival curve by time (in days) |

Table E3: Patient outcome metrics sorted in descending order of group coverage.

## Table E4: Clinical process metrics

| **Metric Number** | **Group count** | **Group coverage** | **Paper count** | **Paper coverage** | **metric** |
| --- | --- | --- | --- | --- | --- |
| 1 | 5 | 36% | 6 | 18% | Intensive care unit (ICU) transfer rate |
| 2 | 2 | 14% | 2 | 6% | Median hours between alert and escalation |
| 3 | 1 | 7% | 1 | 3% | ICU transfer after 1st alert |
| 4 | 1 | 7% | 1 | 3% | Transfer to nursing home or long-term acute care |
| 5 | 1 | 7% | 1 | 3% | Adjusted relative risk for ICU admissions within 30 days after alert |
| 6 | 1 | 7% | 1 | 3% | Escalation to stepdown or ICU |
| 7 | 1 | 7% | 1 | 3% | Escalation to ICU within 12hrs |
| 8 | 1 | 7% | 1 | 3% | Escalation to ICU within 24hrs |
| 9 | 1 | 7% | 1 | 3% | Primary care team physician called by Rapid Response Team (RRT) nurse |
| 10 | 1 | 7% | 1 | 3% | Number of new code status order |
| 11 | 1 | 7% | 1 | 3% | Palliative care consult ordered following alert |
| 12 | 1 | 7% | 1 | 3% | Patients who died with no care consult ordered prior to death |
| 13 | 1 | 7% | 1 | 3% | Observed-to-expected mortality (on-going reporting) |
| 14 | 1 | 7% | 1 | 3% | Any order within 6hrs of alert |
| 15 | 1 | 7% | 1 | 3% | Medication order within 6hrs of alert |
| 16 | 1 | 7% | 1 | 3% | Cardiovascular support ordered within six hours of an alert |
| 17 | 1 | 7% | 1 | 3% | Lactate ordered within 2hr of fist alert |
| 18 | 1 | 7% | 1 | 3% | Required mechanical ventilation |
| 19 | 1 | 7% | 1 | 3% | Time to intervention: defined as the interval between onset of patient deterioration and any order placed for that patient |
| 20 | 1 | 7% | 1 | 3% | Time to response: defined as the interval between onset of patient deterioration and administration of medication, fluids, or supplemental oxygen. |
| **Diagnostic & therapeutic interventions administered within N (e.g. 24h) of alert** | | | | | |
| 21 | 1 | 7% | 1 | 3% | antibiotics |
| 22 | 1 | 7% | 1 | 3% | Antiarrhythmics |
| 23 | 1 | 7% | 1 | 3% | Anticoagulants |
| 24 | 1 | 7% | 1 | 3% | Diuretics/antihypertensives |
| 25 | 1 | 7% | 1 | 3% | Bronchodilators |
| 26 | 1 | 7% | 1 | 3% | Anticonvulsives |
| 27 | 1 | 7% | 1 | 3% | Sedatives/narcotics |
| 28 | 1 | 7% | 1 | 3% | Noninvasive ventilation |
| 29 | 1 | 7% | 1 | 3% | Escalated oxygen support |
| 30 | 1 | 7% | 1 | 3% | Enhanced vital signs, n (%) |
| 31 | 1 | 7% | 1 | 3% | repeat vital signs with 2hr of first alert |
| 32 | 1 | 7% | 1 | 3% | Maintenance intravenous fluids, n (%) |
| 33 | 1 | 7% | 1 | 3% | Vasopressors, n (%) |
| 34 | 1 | 7% | 1 | 3% | Bolus intravenous fluids, n (%) |
| 35 | 1 | 7% | 1 | 3% | Telemetry, n (%) |
| 36 | 1 | 7% | 1 | 3% | Oximetry, n (%) |
| 37 | 1 | 7% | 1 | 3% | New intravenous access, n (% |

Table E4: Clinical process metrics sorted in descending order of group coverage.

# Appendix F: Barrier and Enabler Raw Data

| **Group count (%)** | **Paper count (%)** | **ID** | **Barriers (SALIENT Stage)** | **SALIENT component or element** |
| --- | --- | --- | --- | --- |
| 7 (58%) | 8 (27%) | B01 | Inherent limitations of EHR data, which can be plagued by missingness, inaccuracies, and changes in practice patterns over time; Manually collected vital sign readings resulting in very irregular time series and multiscale gaps. (II+) | DP; AI |
| 5 (42%) | 8 (27%) | B02 | Use of ICU transfer is not a good outcome for AI development and may differ between hospitals. (II/III) | AI; EV |
| 4 (33%) | 5 (17%) | B03 | Alert fatigue (IV/V) | AI; CW; ICA |
| 4 (33%) | 5 (17%) | B04 | Data entry delays, leading to delayed predictions (III+) | DP |
| 4 (33%) | 4 (13%) | B05 | Differences in prevalence at different sites might require models to be retrained or in the least new thresholds selected for those sites to maintain target PPV. (V) | AI |
| 3 (25%) | 4 (13%) | B06 | Lack of clinician trust. (IV+) | ICA |
| 3 (25%) | 4 (13%) | B07 | Lacking a specific and/or effective action for the clinician to take when alerted; Differential nurse/Doctor role; perceptions of role and value; Variations in hospital governance can be problematic for defining standardised response processes when scaling. (IV+) | CW; ICA; GOV |
| 3 (25%) | 3 (10%) | B08 | Lack of infrastructure to do live EMR data pipelines. (III+) | DP |
| 3 (25%) | 3 (10%) | B09 | Major differences between retrospective data elements and prospective/trial data elements. (III) | DP; AI |
| 3 (25%) | 5 (17%) | B10 | Hard/impossible to assess in an implementation study whether a lack of clinical outcome is due to the algorithm performance or the downstream RRT system; Conducting RCT are often not possible. (IV+) | EV |
| 3 (25%) | 3 (10%) | B11 | Having insufficient event samples to build a model that incorporates patient sub-groups; biases in algorithm for different patient groups. (II) | AI; ethics; QS |
| 2 (17%) | 2 (7%) | B12 | Substantial cost involved for infrastructure, implementation personnel time and ongoing maintenance. (II+) | ICA; GOV |
| 2 (17%) | 2 (7%) | B13 | Lack of individual proficiency of health professionals in the use of hardware and software. (IV+) | CW; ICA |
| 2 (17%) | 2 (7%) | B14 | Didn't measure whether the alerted physician followed through with an action. (IV+) | EV |
| 2 (17%) | 3 (10%) | B15 | Differences in software versions between research environment and production environment. (IV+) | DP; AI |
| 1 (8%) | 1 (3%) | B16 | MLA Retraining concerns: Feedback loops arise when alerts lead to timely treatment. (IV+) | AI |
| 1 (8%) | 1 (3%) | B17 | Alerts dismissed for wrong reasons, e.g., patients with no symptoms or with higher acute complexity. (IV+) | CW |
| 1 (8%) | 1 (3%) | B18 | Alert timing doesn't mate with clinician rounds or shift change. (IV+) | CW |
| 1 (8%) | 1 (3%) | B19 | Problems with documentation leading to adverse outcomes or malpractice exposure. (IV+) | CW; RL; QS |
| 1 (8%) | 1 (3%) | B20 | Staff turnover after implementation. (IV+) | CW; ICA |
| 1 (8%) | 1 (3%) | B21 | AI solution only provides a probability and further assessment is necessary. (IV+) | AI; CW |
| 1 (8%) | 1 (3%) | B22 | No funding for post-implementation surveys etc to evaluate alert fatigue and adoption. (IV+) | EV; CW |
| 1 (8%) | 1 (3%) | B23 | Fluctuating alert scores when continuously provided (III+) | AI; CW |
| 1 (8%) | 1 (3%) | B24 | Change in nursing practice during the duration of the study made it more difficult to observe outcome differences in the current study with the prescribed intervention or evaluate an establish clinical practice. (IV+) | HCI; ICA |

Table F1: Complete listing of barriers identified in the review including the number and percentage (n=12) of groups and the number and percentage (n=30) of papers reporting each barrier. The last column contains the mapping to SALIENT components and elements. Salient components are: HCI=Human computer interface; AI=Artificial intelligence model; CW=clinical workflow; and DP=data pipeline. SALIENT elements are: ICA=Implementation, change management and adoption; EV=Evaluation; RL=Regulatory & legal; QS=Quality & safety; Ethics= privacy, transparency and equity; and GOV=Governance

| **Group count (%)** | **Paper count (%)** | **ID** | **Enablers (SALIENT Stage)** | **SALIENT component or element** |
| --- | --- | --- | --- | --- |
| 8 (61%) | 16 (45%) | E01 | Clinician involvement is essential at all stages of model/HCI development and integration into clinical workflow. (II+) | AI; CW; HCI; GOV |
| 6 (46%) | 13 (37%) | E02 | Identified methods to reduce false alarms & alert fatigue. (II+) | AI; CW; HCI |
| 5 (38%) | 10 (28%) | E03 | Linking the EWS alert to specific clinician actions. Clarifying clinical decision points, who is responsible and the actions to take. (III/IV) | CW; RL |
| 5 (38%) | 7 (20%) | E04 | Using more EMR variables than just vitals can improve accuracy. (II) | AI |
| 4 (30%) | 6 (17%) | E05 | Establish a transdisciplinary team of data scientists, statisticians, hospitalists, intensivists, ED clinicians, RRT nurses, and information technology leaders and develop capabilities across domains. (I+) | ICA; GOV |
| 4 (30%) | 5 (14%) | E06 | Conducting external validations using datasets that are different in both time and geographical location may support models that require less updates & retraining. (II) | AI |
| 4 (30%) | 5 (14%) | E07 | Provide additional data with the alert for clinicians to help contextualise the information. | HCI |
| 3 (23%) | 7 (20%) | E08 | Frequent communications to increase awareness during and after trial e.g., weekly meetings, emails, educational sessions giving progress and setting next goals and highlighting urgent need. (IV+) | ICA |
| 3 (23%) | 5 (14%) | E09 | Iterative approach to design of clinical workflow, human-computer interface and AI model. (II+) | AI; CW; HCI |
| 3 (23%) | 5 (14%) | E10 | Perform post-implementation interview (study) & real-time feedback to identify improvements. (IV+) | CW; HCI; QS; ICA |
| 3 (23%) | 7 (20%) | E11 | Establish a multi-disciplinary governance committee to promote usage, track compliance, provide training and plan for post-trial sustainability; and an external data safety board to oversee safety and AI efficacy. (I+) | GOV; QS |
| 3 (23%) | 6 (17%) | E12 | Staggered deployment across sites. (V+) | ICA |
| 3 (23%) | 3 (8%) | E13 | A “Model Facts” sheet designed to convey relevant information about the model to clinical end users. (II) | ethics; AI; ICA; CW |
| 3 (23%) | 4 (11%) | E14 | Improve model training for imbalanced datasets. (II) | AI |
| 3 (23%) | 5 (14%) | E15 | A silent prospective trial conducted while existing RRT system is in place allows you to independently assess performance of MLA vs existing approach. (III) | EV; ICA; DP |
| 2 (15%) | 4 (11%) | E16 | Conduct improvement initiatives (PDSA) cycles during implementation to quickly garner and act on clinical feedback. (III+) | CW; QS; ICA |
| 2 (15%) | 4 (11%) | E17 | Appoint clinical champions to advocate for the tool. (II+) | ICA |
| 2 (15%) | 2 (5%) | E18 | Implement alternative workflows during peak hours and around staff times. (IV+) | CW; ICA |
| 2 (15%) | 3 (8%) | E19 | Clinicians were taught how to interpret risk scores. (IV+) | CW; ET; ICA |
| 2 (15%) | 4 (11%) | E20 | Strong support from senior leadership. (I+) | ICA; GOV |
| 2 (15%) | 4 (11%) | E21 | Trust in the model increased as the clinician experienced the algorithm make correct predictions and the number of cases that the AI detects that clinicians miss. (IV+) | ICA |
| 2 (15%) | 2 (5%) | E22 | Create a data dictionary to harmonise data for the model across different sites/EHR systems. (V) | DP |
| 2 (15%) | 4 (11%) | E23 | Integrated life care planning (palliative care - built in). (III/IV) | ET; CW |
| 2 (15%) | 3 (8%) | E24 | Incorporating the patient into care decisions; e.g., develop a clinician script to explain to patients why the clinician is suddenly evaluating them. (III/IV) | ET; CW |
| 2 (15%) | 4 (11%) | E25 | Quality tracking post-implementation and sustainability of solution. (IV/V) | EV; QS |
| 2 (15%) | 2 (5%) | E26 | Utilise commonly collected EMR data for the model so that the model is transferable. (II) | DP; AI |
| 1 (7%) | 1 (2%) | E27 | Create a test version of the application to train clinicians and multi-channel training approaches incl. web. (III) | CW |
| 1 (7%) | 1 (2%) | E28 | Visually delineating risk into colors (red cards as high risk, etc). (III) | HCI |
| 1 (7%) | 1 (2%) | E29 | Although numbers and statistical trends were used as evidence, individual patient cases were important to frontline clinicians. (IV+) | ICA |
| 1 (7%) | 1 (2%) | E30 | Efferent arm standardization. (III+) | CW |
| 1 (7%) | 1 (2%) | E31 | Use alarms to produce cognitive change in clinicians from intuition to evidence-based decision making; Use the alerts to uncover clinician biases and re-educate clinicians. (IV+) | CW |
| 1 (7%) | 1 (2%) | E32 | Perform a pre-implementation chart review to identify the key reasons that patients are transferred to ICU. (III) | ICA; CW |
| 1 (7%) | 1 (2%) | E33 | Produce a pre-implementation go-live checklist that must be completed prior to go-live. (III) | CW: HCI |
| 1 (7%) | 1 (2%) | E34 | Develop downtime protocols for when failures occur in the data pipeline. (III) | DP |
| 1 (7%) | 1 (2%) | E35 | Use of standardized guidelines for model development and implementation. (II+) | ethics; AI; GOV |
| 1 (7%) | 1 (2%) | E36 | Use interviews and focus groups to inform the implementation team for changing the clinical workflow. (III) | HCI; CW |
| 1 (7%) | 1 (2%) | E37 | Ensure participants are able and want to be in the early trials. (IV) | CW; ICA |
| 1 (7%) | 1 (2%) | E38 | Integrate system into clinical workflow rather than supplant. (III) | CW; ICA |
| 1 (7%) | 1 (2%) | E39 | Show prediction to providers as soon as they open the chart to help triage. (III) | HCI; CW |
| 1 (7%) | 1 (2%) | E40 | Streamline and standardise documentation to reduce burden on clinicians. (III) | CW |

Table F2: Complete listing of enablers identified in the review including the number and percentage (n=12) of groups and the number and percentage (n=30) of papers reporting each enabler. The last column contains the mapping to SALIENT components and elements. Salient components are: HCI=Human computer interface; AI=Artificial intelligence model; CW=clinical workflow; and DP=data pipeline. SALIENT elements are: ICA=Implementation, change management and adoption; EV=Evaluation; RL=Regulatory & legal; QS=Quality & safety; Ethics= privacy, transparency and equity; and GOV=Governance.

# Appendix G: Outcomes employed by each study

## Table G1: Criteria utilised by papers

| **Criteria ID** | **Criteria specified (with references where applicable)** |
| --- | --- |
| A | Transfer to ICU |
| B | In-hospital death |
| C | Cardiac arrest |
| D | Intubation and mechanical ventilation |
| E | Unplanned return to operating theatre |
| F | Medical emergency team (MET) call |
| G | Use of Oxygen therapy |
| H | Transfer to step-down unit |
| I | Transfer to palliative care |
| J | Issuance of palliative order |
| K | National Early Warning Score 2 (NEWS2) trigger/change |

## Table G2: Clinical deterioration outcome used by each paper

The ID is the identifier of the definition. The logic provides the criteria used, from Criteria in Table G1, and logical operators needed to define the outcome.

| **ID** | **Logic** | **Comments** | **Study IDs** | **Groups** |
| --- | --- | --- | --- | --- |
| [a] | {A or B} |  | Baily et al., 2013  Kollef et al., 2014  O’brien et al., 2019  Mou et al., 2022 | A  A  I  L |
| [b] | {A} |  | Hackmann et al., 2011  Mao et al., 2011 | A  A |
| [c] | {F* or A or E or B**} | * The authors hypothesized that rapid responses trig- gered only by bradycardia, hypopnea, and hyper- tension were frequently not preceded by a gradual deterioration in trends. As a result, MET calls trig- gered by single episodes of hypertension, brady- cardia, or hypopnea in isolation were not included as AEs.  ** unplanned in-hospital death | Bell et al., 2021 | B |
| [d] | {B} |  | Brajer et al., 2020  Ye et al., 2019 | C  N |
| [e] | {E* or C**} | * unexpected; ** lack of palpable pulse with attempted resuscitation | Cho et al., 2020 | D |
| [f] | {C} |  | Kwon et al., 2018  Lee et al., 2021 | D  D |
| [g] | {B or D*} | * Mechanical ventilation for >48hrs | Dziadzko et al., 2018 | E |
| [h] | {A or C*} | * Cardiac arrest on the wards or within 6hrs after ICU transfer, with attempted resuscitation | Kang et al., 2016 | F |
| [i] | {A or C*} | * Cardiac arrest on the wards with attempted resuscitation | Churpek et al., 2014 | F |
| [j] | {[i] or B*} | * death on the ward without activation of the cardiac arrest team | Churpek et al., 2014 | F |
| [k] | {[i] or B*} | * ward death during the post-operative period | Bartkowiak et al., 2019 | F |
| [l] | {A or B or C} |  | Winslow et al., 2022 | F |
| [m] | {A or H} |  | Levin et al., 2022 | G |
| [n] | {A* or B or H*} | * Based on bed movement data. Unexpected transitions were those to a higher level of care such as the ICU or step-down unit, or death | Kia et al., 2020 | G |
| [o] | {A* or B} | * transfer from ward or transitional care unit | Escobar et al., 2012 | H |
| [p] | {A* or B**} | * from ward or transitional care unit. Also: where the patient stayed in the ICU for >6 h or died in the ICU. OR: transfer to the ICU from either the ward or TCU where the patient stayed in the ICU for <6 h if, following this transfer, the next hospital unit was the operating room  ** in a patient whose care directive was ‘‘full code” (i.e., had the patient survived, s/he would have been transferred to the ICU) | Kipnis et al., 2016 | H |
| [q] | {A or B or H or I} |  | Pou-Prom et al., 2022 | J |
| [r] | {[q] or J} |  | Nestor et al., 2020 | J |
| [s] | {A or C* or F**} | * resuscitation call; ** rapid response team (RRT) activation | Romero-Brufau et al., 2021 (both papers) | K |
| [t] | {A or B or D} |  | Singh et al., 2021 | L |
| [u] | {A or G or K} |  | Un et al., 2020 | M |
|  |  |  |  |  |

# Appendix H: Algorithm setpoint decisions

## Table H1: Alert threshold setting basis

| **Paper** | **Target (actual) sensitivity** | **Target (actual) PPV** | **Target (actual) specificity** | **Targeted Alerts per day (actual)** | **Primary objective / comment** |
| --- | --- | --- | --- | --- | --- |
| Bailey et al. 2013 Kollef et al. 2014 | 40 |  | 97.6 | 1-2 alerts per nursing unit/day | Manageable number of alerts |
| Hackmann et al., 2011 Mao et al., 2011 |  |  | 95% |  | Manageable number of alerts per hospital floor per day |
| Bell et al., 2021 |  |  | 98% |  |  |
| Brajer et al. 2020 | 60% | 20% |  | 11.9 alerts/day/100 patients  6 alerts/day/100 patients | Revised and used PPV rather than sensitivity to set alert |
| Kwon et al. 2018 | Yes |  |  |  | Sensitivity vs mean alarm count per day |
| Lee et al., 2021 |  |  |  |  | High sensitivity, low false alarm rate. |
| Dziadzko et al., 2018 | (63%) | (21%) |  |  |  |
| Kang et al., 2016 Churpek et al., 2014 | (60%) |  | 95% |  |  |
| Winslow et al., 2022 |  |  |  | 10 patients with red scores/day across the four-hospital system. |  |
| Levin et al., 2022 | (40%) | (13%) | (0.78%) | eight primary team and six RRT alerts / day (14 alerts total, seven per intervention unit) |  |
| Martinez et al., 2022 |  |  |  |  | clinically sustainable alert frequency, minimize clinical burden & alert fatigue |
| Kipnis et al., 2016 Escobar et al., 2020 | (49%) |  |  | one new alert /day / 35 patients |  |
| Lisk et al., 2020 |  |  |  |  | mitigate the highest risk and not generate alert fatigue |
| Drummett et al., 2016 | (25%) |  | (98%) |  | a level of workload that was felt to be acceptable by clinicians |
| O’Brien et al., 2019 |  | 10% (red) |  |  |  |
| Pou-Prom et al., 2022 Nestor et al., 2020 Verma et al., 2021 | (50%) | 40% |  |  | clinicians expressed the need to minimize false alerts, and they recommended a ratio of 2 false alerts to a single true positive |
| Romero-Brufau et al., 2021 |  |  |  | 1 alert /day/10 patients | To reduce risk of alert fatigue |

# Appendix I: Evaluation Stages by Group

## Table I1: Evaluation Stages (SALIENT stage) by Group

| **Paper** | **Group** | **Stage II: Retrospective** | **Stage III: Prospective / Real-time (Length in Months)** | **Stage IV: Trial (Length in Months)** |
| --- | --- | --- | --- | --- |
| Mao et al., 2011  Hackmann et al., 2011  Bailey et al., 2013  Kollef et al., 2014 | A | Yes  Yes  Yes | 3 | 12  4 |
| Bell et al., 2021 | B | Yes | 6 |  |
| Brajer et al., 2020 | C | Yes | 2 |  |
| Kwon et al., 2018  Cho et al., 2020  Lee et al., 2021 | D | Yes  Yes |  |  |
| Dziadzko et al., 2018 | E | Yes |  |  |
| Churpek et al., 2014  Churpek et al., 2014  Kang et al., 2016  Bartkowiak., 2019  Winslow et al., 2022 | F | Yes  Yes  Yes | 4.5 | 2 |
| Kia et al., 2020  Levin et al., 2022 | G | Yes | 0.5 | 8 |
| Escobar et al., 2012  Kipnis et al., 2016  Drummett et al., 2016  Escobar et al., 2016  Granich et al., 2016  Escobar et al., 2020  Lisk et al., 2020  Paulson et al., 2020  Martinez et al., 2022 | H | Yes  Yes |  | 24  (as above) |
| O’brien et al., 2019 | I | Yes | 2.5 |  |
| Nestor et al., 2020  Verma et al., 2021  Pou-Prom et al., 2022 | J | Yes  Yes | 10 | 20 |
| Romero-Brufau et al., 2021 | K | Yes | 4 | 10 |
| Singh et al., 2021  Mou et al., 2022 | L |  | 2 |  |
| Chen et al., 2019  Un et al., 2020 | M |  |  | 1 |
| Ye et al., 2019 | N | Yes | 9 |  |
| Minimum  Average  Maximum  Number of groups (%)  % of groups that trialed | 14 | 12 (86%)  86% | 0.5  4.4  10  10 (71%)  71% | 1  10.1  24  7 (50%) |
